# Predicting the growth performance of growing-finishing pigs based on net energy and digestible lysine intake using multiple regression and artificial neural networks models

**DOI:** 10.1186/s40104-022-00707-1

**Published:** 2022-05-13

**Authors:** Li Wang, Qile Hu, Lu Wang, Huangwei Shi, Changhua Lai, Shuai Zhang

**Affiliations:** State Key Laboratory of Animal Nutrition, College of Animal Science and Technology, China Agricultural University, Beijing, 100193 P. R. China

**Keywords:** Multiple regression model, Neural networks, Pig, Prediction

## Abstract

**Backgrounds:**

Evaluating the growth performance of pigs in real-time is laborious and expensive, thus mathematical models based on easily accessible variables are developed. Multiple regression (MR) is the most widely used tool to build prediction models in swine nutrition, while the artificial neural networks (ANN) model is reported to be more accurate than MR model in prediction performance. Therefore, the potential of ANN models in predicting the growth performance of pigs was evaluated and compared with MR models in this study.

**Results:**

Body weight (BW), net energy (NE) intake, standardized ileal digestible lysine (SID Lys) intake, and their quadratic terms were selected as input variables to predict ADG and F/G among 10 candidate variables. In the training phase, MR models showed high accuracy in both ADG and F/G prediction (R^2^_ADG_ = 0.929, R^2^_F/G_ = 0.886) while ANN models with 4, 6 neurons and radial basis activation function yielded the best performance in ADG and F/G prediction (R^2^_ADG_ = 0.964, R^2^_F/G_ = 0.932). In the testing phase, these ANN models showed better accuracy in ADG prediction (CCC: 0.976 vs. 0.861, R^2^: 0.951 vs. 0.584), and F/G prediction (CCC: 0.952 vs. 0.900, R^2^: 0.905 vs. 0.821) compared with the MR models. Meanwhile, the “over-fitting” occurred in MR models but not in ANN models. On validation data from the animal trial, ANN models exhibited superiority over MR models in both ADG and F/G prediction (*P* < 0.01). Moreover, the growth stages have a significant effect on the prediction accuracy of the models.

**Conclusion:**

Body weight, NE intake and SID Lys intake can be used as input variables to predict the growth performance of growing-finishing pigs, with trained ANN models are more flexible and accurate than MR models. Therefore, it is promising to use ANN models in related swine nutrition studies in the future.

**Supplementary Information:**

The online version contains supplementary material available at 10.1186/s40104-022-00707-1.

## Introduction

To maximize profits in swine production, farmers need to adjust diet formulations and feeding strategies based on their understandings of the relationships between the growth performance of pigs and nutrient supply. However, evaluating the growth performance of pigs in real-time is laborious and expensive. As a result, mathematical models were developed based on easily accessible variables to predict the response variables not easily determined, which has provided an effective approach to quantify the animal production processes and then to improve the efficiency and sustainability of the modern livestock system [1].

Multiple regression (linear or non-linear) is the most convenient tool to model the relationship between response variables and explanatory variables and is commonly used in animal nutrition studies. For example, the diet characteristics (e.g., available energy values in swine diets) [2] or production performance of livestock (e.g., milk yield in dairy cow) [3] could be predicted using multiple regression (MR) models with relatively high accuracy. The prerequisite for MR utilization is assuming a regression relationship (linear or non-linear) between the response variables and the explanatory variables, however, in reality, the relationships among variables are usually complex, resulting in large predictive errors in some situations when modelling using MR, e.g. modelling maintenance energy requirement of pigs [4, 5]. Therefore, more efficient mathematical tools are needed to be evaluated if they could better model the complicated animal production systems to achieve better predictive performance.

As the integration of the information science and other disciplines in recent years, artificial neural networks (ANN) models were introduced into agriculture research considering their capacity to deal with complex and flexible non-linear interrelationships without prior assumptions [6]. The ANN model has a parallel and distributed information processing structure, which consists of interconnected processing elements (artificial neurons or nodes), thus is more suitable to quantify the unknown or very complex relationships. Moreover, as a supervised learning process, ANN models usually have stronger learning ability and higher fault tolerance than MR models [7]. Recently, ANN models were reported to exhibit better prediction performance than MR models in other disciplines [8–11]. However, in swine research, the application of ANN models mainly focused on image identification, behaviour detection and disease detection. Only a few visionary scientists have applied ANN models in swine nutrition research, e.g. Ahmadi and Rodehutscord conducted a preliminary work using ANN models to predict metabolizable energy (ME) values in pig feed [12]. Thus, more works can be done to extend the applications of ANN models in swine nutrition.

To our knowledge, no previous studies have reported the utilization of ANN models in predicting the growth performance of pigs. Therefore, it is unclear whether ANN was still more powerful than MR models in predicting pigs’ growth performance. Therefore, the objectives of this study were to 1) predict the average daily gain (ADG) and feed conversion ratio (F/G) of growing-finishing pigs based on dietary nutrient intake by developing MR models and ANN models 2) compare the performance of the two models in growth performance prediction in pigs.

## Materials and methods

**Fig. 1:**
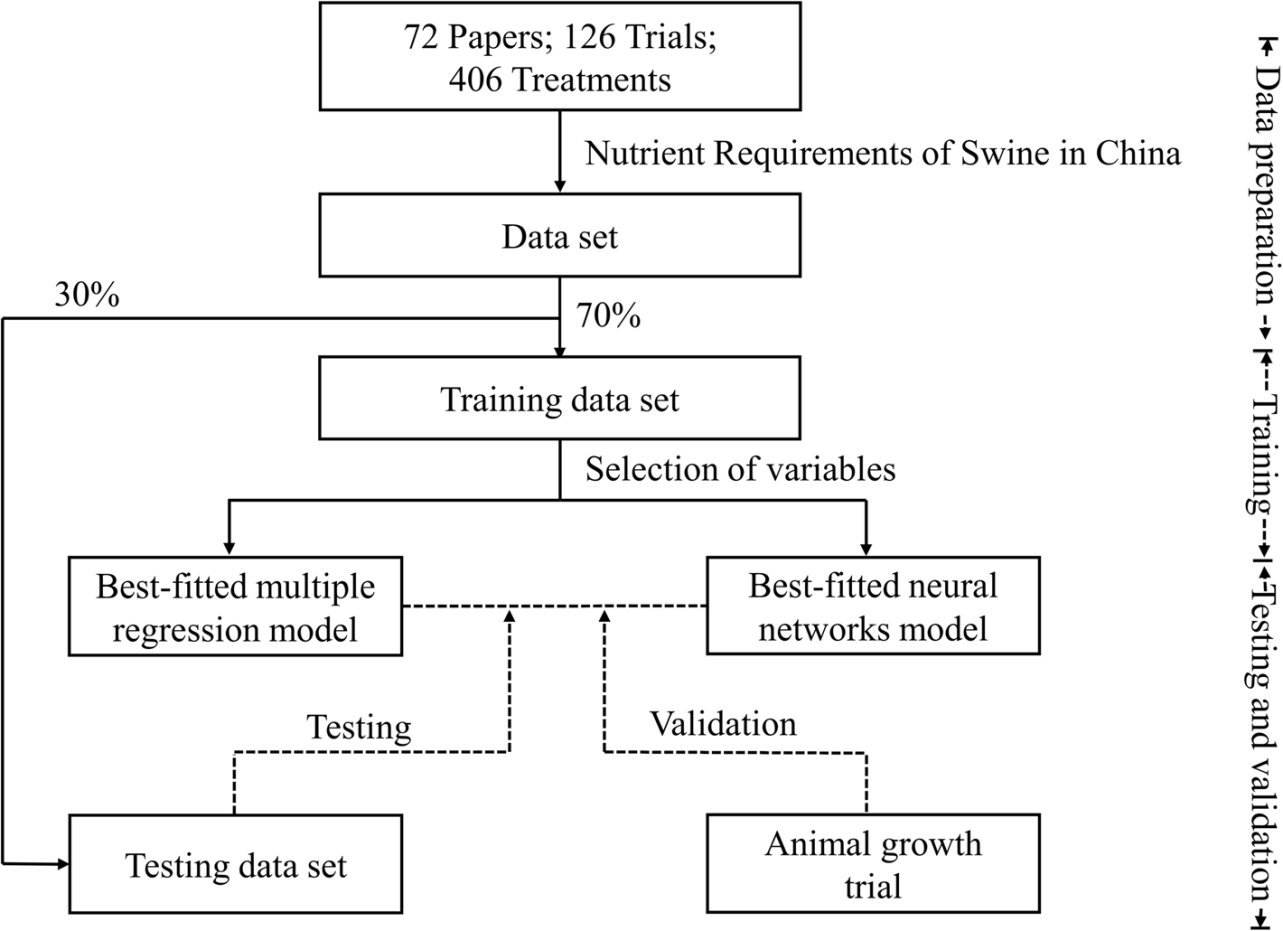
The general scheme of this study.

The general scheme of this study was outlined in Fig. 1.

### Data sources

Data were derived from peer-reviewed journal articles published from 2010 to 2019 using the Web of Science online database. Considering the changes in the genetic background due to the progress in pig breeding, data from earlier literature were not considered. The keywords and phrases used for literature research were “pig OR pigs OR swine AND growth performance”, and 212 papers with 285 trials and 1170 treatment diets were collected after screening.

According to our research objectives, the final database articles were selected based on the following criteria: (i) belongs to research articles published in English; (ii) included control treatment with adequate replicates per treatment (≥ 6) and proper randomization of treatments, and pigs used in the trial had ad libitum access to feed and water; (iii) presented complete diet compositions with ingredients included in Nutrient Requirements of Swine in China [13], and reported the growth performance data (body weight gain, feed intake, or feed conversion ratio) of pigs. Moreover, treatment diets included effects of antibiotics or feed additives, or not formulated based on corn and soybean meal, or used intact males, immunocastrated males, or pigs fed Ractopamine HCl were excluded from the database. Clear segmentation of pig breeds would produce more accurate input data and ultimately a more accurate prediction. Consequently, only Duroc × Landrace × Yorkshire crossbred pigs were included to eliminate the effects of genetic background. The experimental period should keep in the range of 7 d to 35 d in order that the calculated average BW can represent the growth stages of pigs. In addition, all the dietary nutrient concentrations of the diet should be given at least 85% of the recommended of NRC [14]. Finally. the Explore Outliers procedure in JMP Pro version 14.0 (SAS Institute, Cary, NC, USA) was used to eliminate the outliers. After excluding trials using the above criteria, 126 trials and 406 treatments were fetched from 72 papers for further analysis. The papers used in this study were given in Additional file 1: Table S1  and the statistic information of the training data set and testing data set were given in Additional file 1: Table S2.

### Datasets preparation

Growth performance data extracted from the selected papers were recorded in a template that included ADG, average daily feed intake (ADFI), and F/G of pigs for each treatment diet. If any parameter above was missing, it was calculated from the other reported parameters in the paper if available, otherwise, the whole record (treatment diet) was discarded. The average BW of pigs fed each treatment diet was calculated by averaging the initial and final BW of all pigs in the same treatment group.

Nutrient concentrations of each treatment diet were calculated based on the nutrient concentrations of each ingredient and its proportion in the diet, and nutrient concentrations of individual ingredients from the Nutrient Requirement of Swine in China [13] were used as the reference values. Net energy (NE) was chosen because it is considered the most accurate system to quantify the energy content in pig feed currently [15]. All amino acids were expressed as the standardized ileal digestible (SID) concentrations (AA contents in ingredients multiplying the corresponding standardized ileal digestibility of the AA) to overcome the disadvantages and limitations of apparent ileal digestibility (AID) and true ileal digestibility (TID) [16]. The nutrient intakes were calculated by multiplying ADFI by nutrient concentrations of the corresponding treatment diet. The specific nutrient intake variables included in the original dataset were: NE intake (kcal/d), CP intake (g/d), SID lysine intake (g/d), SID methionine intake (g/d), SID threonine intake (g/d), SID tryptophan intake (g/d), SID valine intake (g/d), acid detergent fiber intake (ADF, g/d) and neutral detergent fiber intake (NDF, g/d) on an as-fed basis.

Then the growth performance and nutrient intake data from the 406 treatment diets were randomly split into a training data set containing 70% of the observations and a testing data set containing the remaining observations. Descriptive statistics of the variables in training and testing data sets were presented in Table 1.

**Table 1:** Descriptive statistics of variables on pig growth performance and dietary nutrient concentrations used in developing the prediction models^1^

Variables	Training data set						Testing data set				
	Unit	*n*	Range	Mean	SD	Median	*n*	Range	Mean	SD	Median
BW	kg	287	5.5-118.6	45.6	36.7	37.0	119	5.6-116.1	41.8	33.2	35.8
ADG	g/d	287	142-1200	634	268	697	119	165-1060	647	252	680
ADFI	g/d	287	259-3667	1600	1058	1550	119	269-3575	1543	974	1450
F/G	-	287	1.16-4.35	2.26	0.82	2.08	119	1.21-3.85	2.17	0.72	1.97
NE intake	kcal/d	287	602-9906	4168	2790	3958	119	719-9415	4015	2573	3687
CP intake	g/d	287	40-646	242	143	224	119	58-684	244	138	232
SID Lys intake	g/d	287	3.30-57.74	14.20	7.66	14.61	119	4.13-32.19	14.27	6.64	13.75
SID Met intake	g/d	287	0.68-23.07	5.46	3.05	5.16	119	1.12-18.55	5.51	3.00	4.83
SID Thr intake	g/d	287	1.13-24.05	8.49	4.64	9.29	119	1.99-23.26	8.52	4.50	7.66
SID Try intake	g/d	287	0.27-10.75	2.47	1.39	2.53	119	0.56-7.54	2.49	1.28	2.24
SID Val intake	g/d	287	1.58-27.84	10.49	5.98	10.85	119	2.52-27.96	10.55	5.71	9.73
ADF intake	g/d	287	4-240	55	42	55	119	4-247	56	43	49
NDF intake	g/d	287	15-604	185	135	176	119	15-431	177	120	164

*ADF* acid detergent fiber, *ADFI* average daily feed intake, *ADG* average daily gain, *BW* body weight, *CP* crude protein, *F/G* feed conversion ratio, *NDF* neutral detergent fiber, *NE* net energy, *SD* standard deviation, *SID* standardized ileal digestible.

^1^ Data were collected from 72 peer-reviewed articles published from 2009-2019 with 406 treatment means. All the dietary nutrient concentrations were re-calculated based on reported diet formulations in the peer-reviewed articles and the nutrient compositions of ingredients published in Nutrient Requirements of Swine in China [13].

### Variables selection

Theoretically, more input variables indicate increased discriminative power of the predictive models, but adding irrelevant variables can also distract the learning algorithm and defect the predictive performance [17]. Thus, the Fit Model procedure with standard least squares personality and emphasis on Effect Screening function in JMP Pro version 14.0 was firstly used to eliminate excess variables on ADG and F/G prediction. The input variables included the BW and all the nutrient intake parameters, as well as their interactive effects, and *P* < 0.05 was used as a selection criteria. Since no significant interactive effects were detected, the quadratic and cubic terms of the selected input variables were further included in the MR models, and the improved R^2^ of each model was regarded as the selection criteria.

### Developing MR models using training data set

The Fit Model procedure with Stepwise Regression personality in JMP Pro version 14.0 was used to establish MR models to predict ADG or F/G. The NE intake (kcal/d), SID Lys intake (g/d), BW and their quadratic terms within each treatment diet (287 observations) in the training data set were treated as predictors for model development, and study effects were included as a random effect. The mixed direction and *P*-value Threshold stopping rules were chosen and the variables are entered and removed from the model at a probability below 0.01. Models with the maximal R^2^, minimized Akaike information criterion (AIC) and Bayesian information criterion (BIC) were identified as the best-fitted MR model [18], which was then checked through graphical inspection for normality on the residuals [19].

### Developing ANN models using training data set

Artificial neural networks are programs designed to learn and process information by simulating the human brain, which consists of three main components: an input layer, a series of hidden layers and an output layer [20]. The number of hidden layers in ANN is dependent on the complexity of the relationships between inputs and target outputs. More hidden layers can increase the chance of obtaining local minima during the training phase and contribute to a more unstable gradient. Neurons, or called nodes, are the basic unit to compose hidden layers, which receive input from the input layer, scale each input by a weight, add a bias and then apply an activation function to the result [21]. The structure of a classical feedforward ANN model can be demonstrated using the following mathematics formulations:

$$ {H}_1=\sum {I}_m\times {w}_m+{a}_m\kern0.5em \mathrm{and}\kern0.5em {O}_1={F}_{activation}\left({H}_n+{b}_n\right) $$

where *H*_1_ was the value in the 1st node in the hidden layer, *I*_*m*_ was the value of the *m*th input variable, *w*_*m*_ was the weighting factor between the *m*th input variable and the 1st node in the hidden layer, *a*_*m*_ was the bias; *O*_1_ was the value of the 1st output variable, *H*_*n*_ was the value of the *n*th node, *b*_*n*_ was the bias, and *F*_*activation*_ was the activation function.

The Neural Network procedure in JMP Pro version 14.0 was used to develop a series of ANN models and the details were presented later. In the current study, the three-layer ANN, using Scaled Conjugate Gradient algorithm, including one input layer, one hidden layer and one output layer, was used for model development. Variables used in ANN models were the same as those in MR models to ensure the comparability between models. Moreover, it is necessary to normalize the data used in establishing the ANN models to get prediction errors with step sizes and update systematic weights due to the different unit scales the input variables have [22]. The training data set was normalized using the min-max approach as follows:

$$ {x}_i^{\prime }=\frac{x_i-\mathit{\min}(x)}{\mathit{\max}(x)-\mathit{\min}(x)} $$

where *x*_*i*_ was the observed value of the *i*th input data and ${x}_i^{\prime }$ was the *i*th normalized data.

The output layer included two variables: ADG and F/G. Because the input variables were normalized, the predicted output values were re-scaled using the minimal and maximal values of the training data for model evaluation. The re-normalization was conducted as follows:

$$ {y}_i={y}_i^{\prime}\times \left(\mathit{\max}(y)-\mathit{\min}(y)\right)+\mathit{\min}(y) $$

where *y*_*i*_ was the predicted value of the *i*th output and ${y}_i^{\prime }$ was the *i*th normalized output predicted using the ANN model.

The training conditions including a learning rate of 0.1, training epochs of 1000, and the Squared penalty method were adopted in the current study. Karlik et al. [23] compared five different activation functions and found hyperbolic tangent function performs better recognition accuracy than the other four functions. Meanwhile, Radial basis function neural networks is one of the most popular neural network architectures [24]. Thus, the hyperbolic tangent function ($\mathit{\tanh}(x)=\frac{{\mathrm{e}}^{2x}-1}{{\mathrm{e}}^{2x}+1}$) and radial basis function ($RB(x)={e}^{-{x}^2}$) were chosen as candidate activation functions between the hidden layer and the output layer. Identifying the optimal number of neurons in the hidden layer is also a major step for establishing ANN models [25], so the mono-hidden layer structure containing 1 to 10 nodes were evaluated.

Models with different nodes and activation functions were selected by the R^2^ and root mean square error (RMSE) and the model with the maximal R^2^ and minimized RMSE was considered as the best-fitted ANN model.

### Comparison between the MR models and the ANN models using testing data set

The testing data set was used to generate predicted ADG and F/G values based on the best-fitted MR models developed using the training data set. Meanwhile, the same testing data set was normalized, input into the best-fitted ANN models, and then re-scaled using the re-normalization equation to generate another group of predicted ADG and F/G values.

The RMSE, R^2^ and concordance correlation coefficients (CCC) were calculated using the two groups of prediction data to evaluate the performance of the selected MR models and ANN models:

$$ RMSE=\sqrt{\frac{1}{n}\sum \limits_{i=1}^n{\left({y}_i-{y}_i^{\prime}\right)}^2} $$

$$ {R}^2=1-\frac{\sqrt{RMSE}}{S_Y^2} $$

$$ CCC=\frac{2r{S}_Y{S}_{Y^{\prime }}}{{\left(\overline{y}-{\overline{y}}^{\prime}\right)}^2+{S}_Y^2+{S}_{Y^{\prime}}^2} $$

where *y*_*i*_, ${y}_i^{\prime }$, ${S}_Y^2$ and ${S}_{Y^{\prime}}^2$ were the predicted output values using MR model and ANN model and their corresponding variables, respectively. The lower RMSE value and higher R^2^ and CCC values were considered as an indicator of better accuracy.

The observed vs. predicted plots were generated using observed values and predicted values from MR models or ANN models, and the following linear equation was obtained in each plot:

$$ y=a+ bx $$

where *x* refers to the observed growth performance variable (ADG or F/G), *y* refers to the predicted variable. The plot with a slope closer to 1 represents better prediction performance of the corresponding model.

### Experimental design of the animal trial used to validate the prediction models

An animal trial was conducted to collect data for further comparison between the MR models and the ANN models. The animal handling procedures received approval from the Animal Care and Use Ethics Committee of the China Agriculture University (Beijing, China).

One hundred and ninety-two Duroc × Landrace × Yorkshire crossbred pigs with an average initial body weight of 35.29 ± 3.11 kg were randomly assigned to 4 treatment diets in a completely randomized design, with 4 replicate pens per treatment and 12 replicate pigs (6 barrows and 6 gilts) per pen. The experiment design was a 2 × 2 factorial with respective factors being two levels of SID Lys (100% Lys requirement vs. 130% Lys requirement) and two levels of NE (100% NE requirement vs. 105% NE requirement) content in diets (Additional file 1: Table S3). All the diets were fed in mash form and were formulated to meet the nutrient requirement of pigs [13]. The animal trial lasted for 84 d, and the individual pig BW and feed consumption (on pen basis) were measured on d 0, 14, 28, 42, 56, 70, 84 to calculate the ADG and F/G. Nutrient intakes of each pen were calculated using nutrient profiles presented in the Nutrient Requirements of Swine in China [13] and the ADFI of each pen. The values of pig BW, NE intake (kcal/d), SID Lys intake (g/d) and their quadratic terms of each pen are considered as one observation. In total, 96 observations were extracted from 4 replicates of 4 treatments and 6 phases. The details of each observation obtained from the animal trial were presented as Additional file 1: Table S4.

### Comparison between the MR models and the ANN models using validation data set gained from the animal trial

The validation data set gained from the animal trial was used to generate predicted ADG and F/G values based on the best-fitted MR models and the best-fitted ANN models established in the training phase. Again, all the input data were normalized firstly, and the output data were re-scaled lastly when the ANN models were applied as described in the previous part. The observed vs. predicted plots were generated as described previously.

Based on the results of previous steps, MR models exhibited larger errors at greater BW range of pigs. To further check the hypothesis whether growth stages would influence the prediction performance of the two models, the mean absolute error (MAE, $\mathrm{MAE}=\frac{1}{n}\sum \limits_{i=1}^n\left|{y}_i-{y}_i^{\prime}\right|$) between the observed variables and the predicted variables from the MR models or ANN models were calculated. The MAE values of the two kinds of models were grouped based on pig BW with a 10 kg interval as follows: 40-50 kg, 50-60 kg, 60-70 kg, 70-80 kg, 80-90 kg, 90-100 kg and 100-110 kg. Two-way ANOVA was conducted with predictive method and growth stage as the major effects. *P* < 0.05 was considered as significantly different and 0.05 ≤ *P* ≤ 0.10 was considered as a significant tendency.

## Results

### Variables selection

The results of the two-step variable selection were shown in Table 2. Among the ten candidate variables, pig BW, NE intake and SID Lys intake showed the minimized *P*-value, which were all below 0.01. The MR models generated using those three variables in linear, quadratic, and cubic terms had shown R^2^ of 0.89, 0.93, and 0.93 in ADG prediction, and 0.87, 0.89, and 0.88 in F/G prediction, respectively. Therefore, BW, NE intake, SID Lys intake and their quadratic forms were chosen as the input variables for the following model development.

**Table 2:** Selection of input variables^1, 2^

Step 1			Step 2^3^	
Variables	*P*-value	Selection	Forms^3^	Selection
BW	<0.01	√	Linear	√
NE intake	<0.01	√	Quadratic	√
CP intake	0.30		Cubic	
SID Lys intake	<0.01	√		
SID Met intake	0.29			
SID Thr intake	0.10			
SID Trp intake	0.37			
SID Val intake	0.11			
ADF intake	0.29			
NDF intake	0.21			

*ADF* acid detergent fiber, *ADFI* average daily feed intake, *ADG* average daily gain, *BW* body weight, *CP* crude protein, *F/G* feed conversion ratio, *NDF* neutral detergent fiber, *NE* net energy, *SID* standardized ileal digestible

^1^ Step 1 was used to select the most sensitive variables to predict ADG and F/G, and Step 2 aimed to find the appropriate forms of input variables.

^2^ No significant interactive effects of the selected variables in Step 1 were detected, with *P*-value > 0.05.

^3^ The R^2^ for predicting ADG in different forms were: linear: 0.89; quadratic: 0.93; cubic: 0.93. The R^2^ for predicting G/F in different forms were: linear: 0.87; quadratic: 0.88; cubic: 0.88.

### Best-fitted MR models

The best-fitted MR models for predicting ADG and F/G were presented in Table 3. For ADG prediction, the MR model using BW, SID Lys intake, SID Lys intake^2^, NE intake, and NE intake^2^ exhibited the smallest AIC (AIC = 3278), BIC (BIC = 3381), RMSE (RMSE = 72) and the maximized R^2^ (R^2^ = 0.929). Pig BW, SID Lys intake^2^, and NE intake^2^ had negative effects on ADG while SID Lys intake and NE intake had a positive effect on ADG. For F/G prediction, the MR model using BW and BW^2^, SID Lys intake, and NE intake had the smallest AIC (AIC = 92), BIC (BIC = 116), RMSE (RMSE = 0.28) and the maximized R^2^ (R^2^ = 0.886). The BW, BW^2^, and NE intake had positive effects on F/G while SID Lys intake had an adverse effect on F/G.

**Table 3:** Best-fitted MR models developed in the current study to predict growth performance of growing-finishing pigs^1^

Performance	Models^2^	R^2^	AIC	BIC	RMSE
ADG	= 57 - 1.63 × BW + 25.42 × SID Lys - 0.360 × SID Lys^2^ + 0.120 × NE - 4.630 × 10^-6^ × NE^2^	0.929	3278	3381	72
F/G	= 1.31 + 1.955 × 10^-2^ × BW + 9.064 × 10^-5^ × BW^2^ - 4.764 × 10^-2^ × SID Lys + 2.10 × 10^-4^ NE	0.886	92	116	0.28

*ADG* average daily gain, *AIC* akaike information criteria, *BIC* bayesian information criteria, *BW* body weight, *F/G* feed conversion ratio, *NE* net energy, *RMSE* root mean square error, *SID Lys* standardized ileal digestible lysine.

^1^ The SID Lys and NE in the equations were the SID Lys intake and NE intake.

^2^ The variables in the equations were selected by a *P*-value < 0.01. Both the best-fitted MR models were generated using the training data set (*n* = 287).

**Fig. 2:**
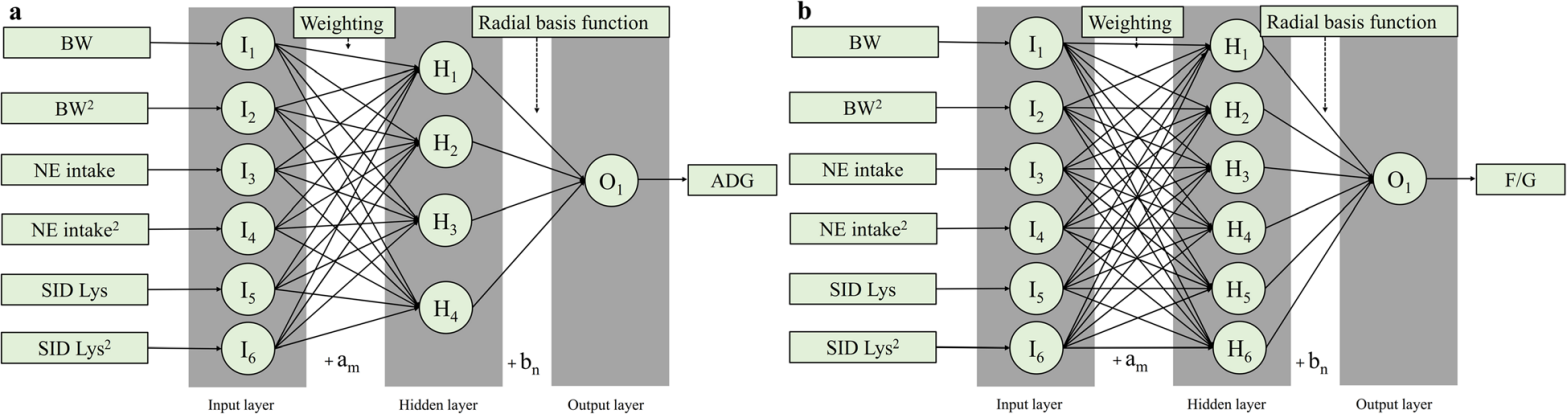
The response of ADG on different SID Lys intake (**a**) and NE intake (**b**). The curves were generated by the best fitted MR models in training. Only SID Lys intake and SID Lys intake^2^ were considered as input variables in Fig. 2a while other variables were neglected. Only NE intake and NE intake^2^ were considered as input variables in Fig. 2b.

To better clarify the inconsistence between linear form and quadratic form of SID Lys and NE intake on their contributions to ADG, the responses of ADG on varied SID Lys or NE intake levels were illustrated in Fig. 2. It should be pointed out that Fig. 2 considered the single contribution of SID Lys or NE intake on ADG but ignored the influence of other factors. It was indicated that ADG increased with greater SID Lys intake level only when the SID Lys intake was below 38 g/d. Moreover, the improvement of ADG was observed as the NE intake increased within the range of 0-10,000 kcal/d.

### Best-fitted ANN models

**Fig. 3:**
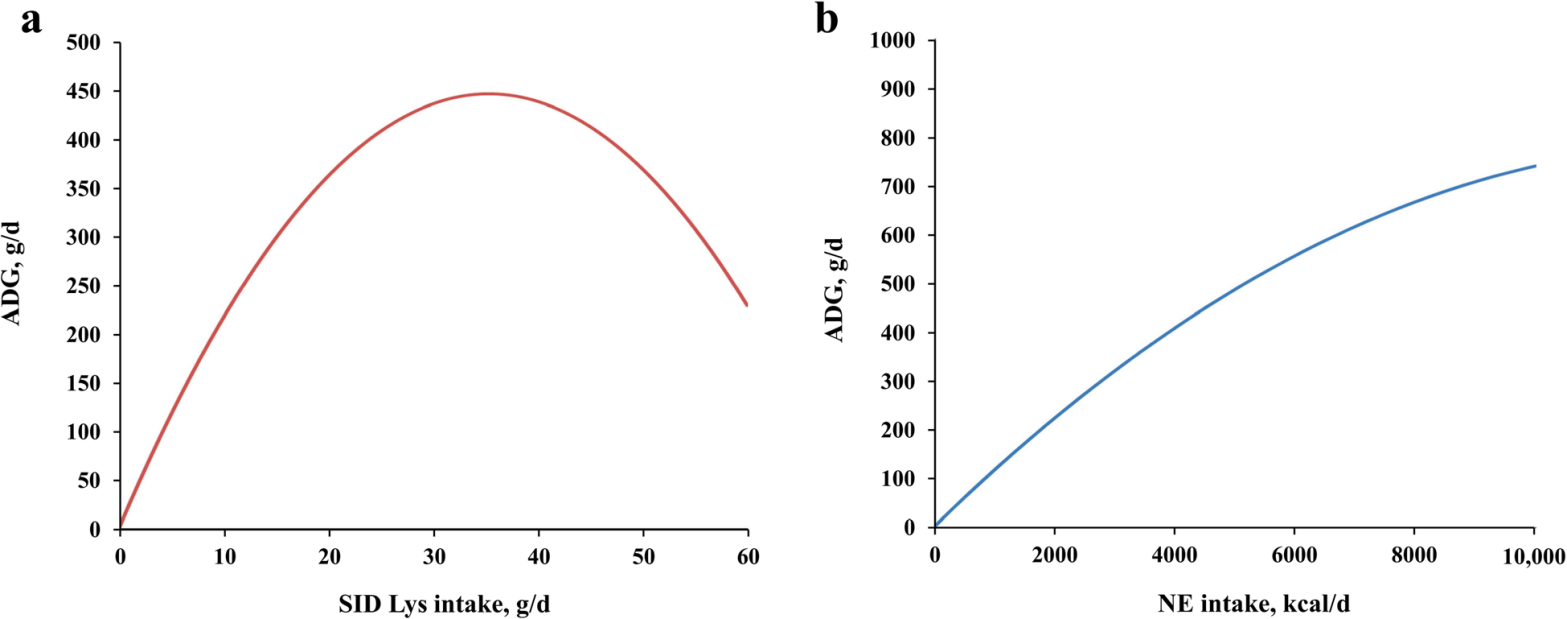
The structure of the best-fitted artificial neural networks in predicting ADG (**a**) and F/G (**b**). *H*_1_ was the value in the 1st node in the hidden layer; *I*_1_ was the 1st input; *a*_*m*_ was the bias; *O*_1_ was the value of the 1st output variable; *H*_1_ was the value of the 1st node; *b*_*n*_ was the bias; *F*_*activation*_ was the activation function.

The structures of the two best-fitted ANN models for predicting ADG and F/G were presented in Fig. 3. The predictive performances on ADG and F/G of ANN models with different neurons in 1 hidden layer using different activation functions were exhibited in Tables 4 and 5. The best-fitted ANN models for ADG and F/G prediction were those using radial basis function with 4 and 6 nodes, with R^2^ of 0.925 and 0.905, and RMSE of 51 and 21, respectively.

**Table 4:** The performance of ANN models with different numbers of nodes and activation functions to predict the ADG of growing-finishing pigs^1^

Number of nodes	Training data set			
	Hyperbolic tangent function		Radial basis function	
	R^2^	RMSE	R^2^	RMSE
1	0.921	75	0.918	77
2	0.932	70	0.936	68
3	0.942	64	0.941	65
4	0.941	65	0.964^*^	51^*^
5	0.952	59	0.958	55
6	0.948	61	0.955	57
7	0.948	61	0.952	58
8	0.942	65	0.945	63
9	0.944	63	0.951	59
10	0.953	58	0.953	58

*RMSE* root mean square error.

^*^ Means the best performance of ANN models with different numbers of nodes and activation functions to predict ADG.

^1^ All the ANN models were generated using the training data set (*n* = 287).

**Table 5:** The performance of ANN models with different numbers of nodes and activation functions to predict the F/G of growing-finishing pigs^1^

Number of nodes	Training data set			
	Hyperbolic tangent function		Radial basis function	
	R^2^	RMSE	R^2^	RMSE
1	0.797	0.37	0.816	0.35
2	0.883	0.28	0.886	0.28
3	0.905	0.25	0.898	0.26
4	0.900	0.26	0.917	0.24
5	0.918	0.23	0.928	0.22
6	0.905	0.25	0.932^*^	0.21^*^
7	0.917	0.24	0.910	0.25
8	0.915	0.24	0.911	0.24
9	0.900	0.26	0.907	0.25
10	0.907	0.25	0.912	0.24

*RMSE* root mean square error

^*^Means the best performance of ANN models with different numbers of nodes and activation functions to predict F/G.

^1^ All the ANN models were generated using the training data set (*n* = 287).

### Comparison between the MR models and the ANN models using testing data set

**Fig. 4:**
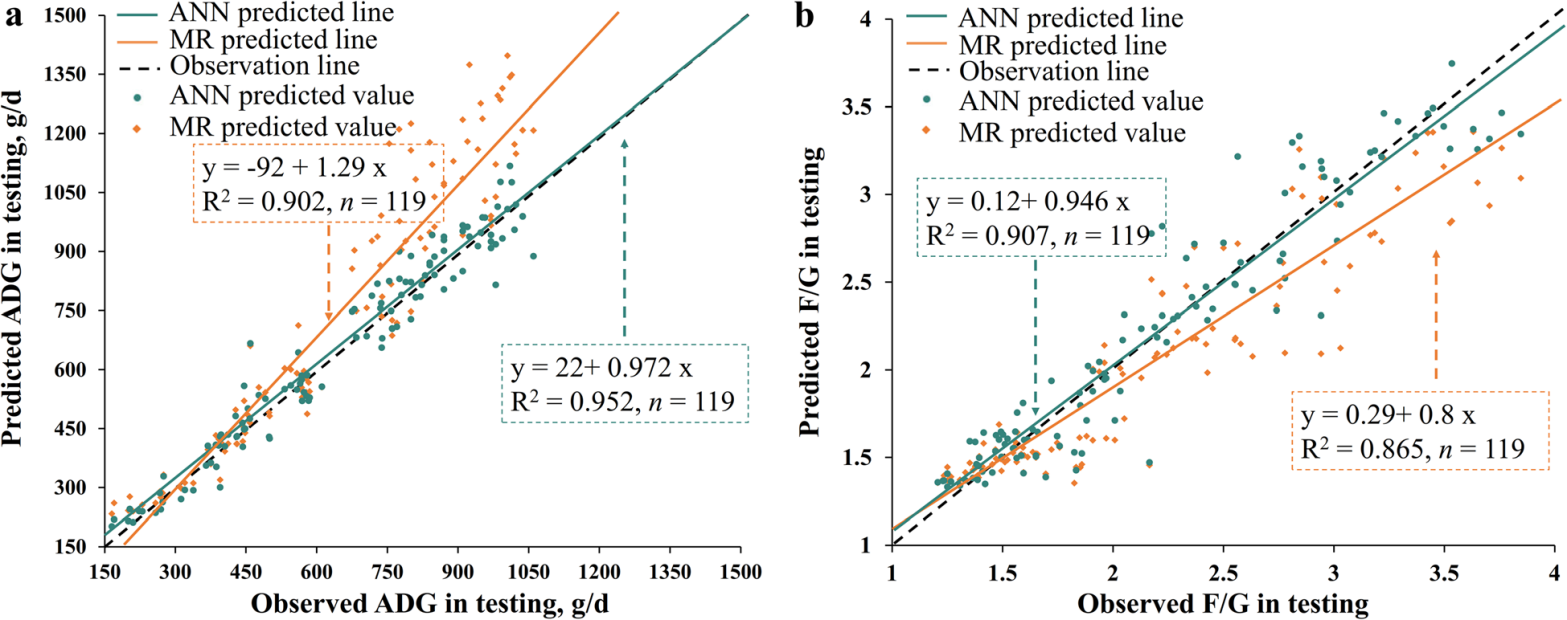
Relationship between the observed vs. the predicted ADG (**a**) or F/G (**b**) from the best-fitted models using testing data set. The best-fitted models were the MR and ANN models generated in training. 119 observations in the testing data set were used in this figure. Each plot represents a sample with observed value and predicted value from prediction models. The green line was the fit line of ANN predicted values while the yellow line was the fit line of MR predicted values. The slope of the fit line which is closer to 1 indicated a lower prediction error of the model.

The comparison between the best-fitted MR models and ANN models using the testing data set was shown in Table 6 and Fig. 4. For both ADG and F/G prediction**,** the ANN models showed lower RMSE and greater CCC and R^2^ values, and had slopes closer to 1 in the observed vs. predicted plots than the MR models, implying greater accuracy of ANN models than MR models.

**Table 6:** Comparison of MR and ANN models using the testing data set

Indicators	ADG	F/G
RMSE		
MR	162	0.30
ANN	55	0.22
CCC		
MR	0.861	0.900
ANN	0.976	0.952
R^2^		
MR	0.584	0.821
ANN	0.951	0.905

*ADG* average daily gain, *CCC* concordance correlation coefficients, *F/G* feed conversion ratio, *RMSE* root mean square error.

In addition, there was a discrepancy in the performance of MR models between the training data set and the testing data set, reflected by a noticeable decrease of R^2^ in ADG prediction (R^2^_training_ = 0.929, R^2^_testing_ = 0.584) and a slight decrease of R^2^ in F/G prediction (R^2^_training_ = 0.886, R^2^_testing_ = 0.821), indicating the occurrence of over-fitting.

### Comparison between the MR models and ANN models using validation data set gained from the animal trial

**Fig. 5:**
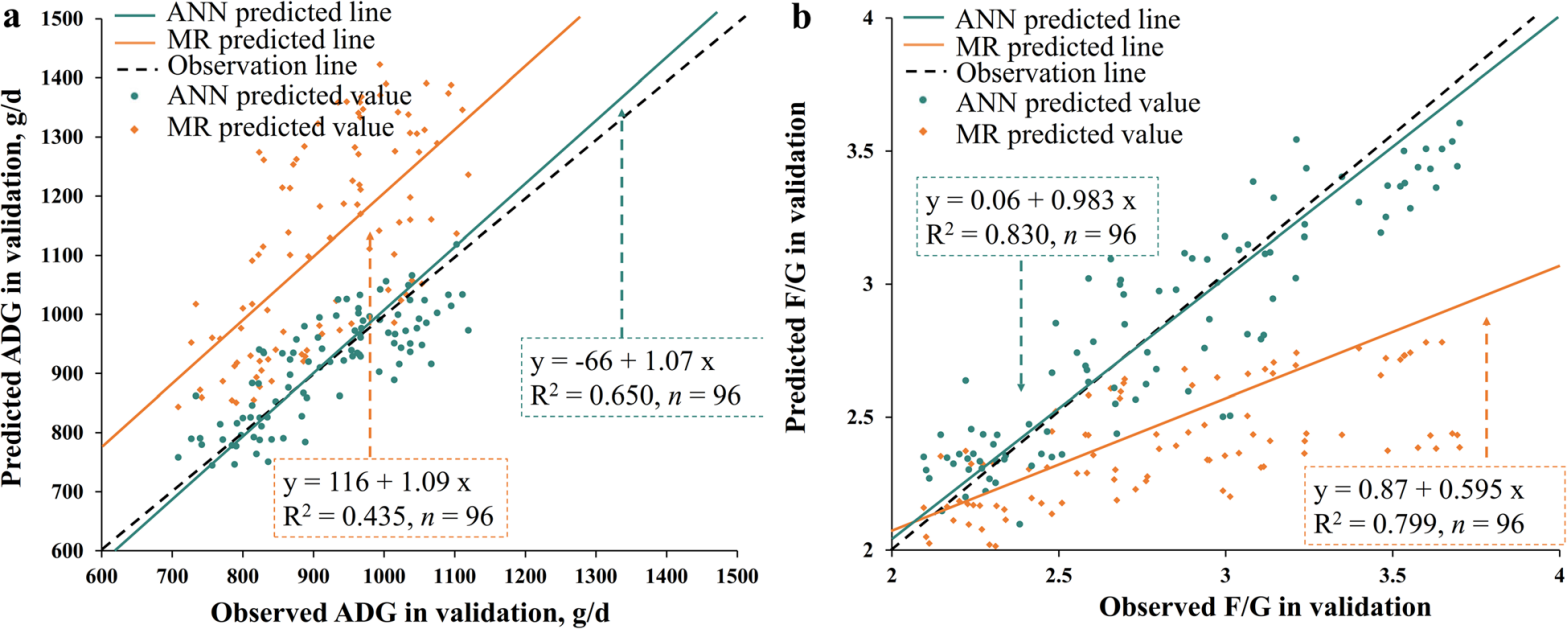
Relationship between the observed vs. the predicted ADG (**a**) or F/G (**b**) from the best-fitted models using validation data set. The best-fitted models were the MR and ANN models generated in training. 96 observations in the animal trial were used in this figure. Each plot represents a sample with observed value and predicted value from prediction models. The green line was the fit line of ANN predicted values while the yellow line was the fit line of MR predicted values. The slope of the fit line which is closer to 1 indicated a lower prediction error of the model.

The comparison between the best-fitted MR models and ANN models using the validation data set gained from the animal trial was shown in Fig. 5. For both ADG and F/G prediction**,** the ANN models showed slopes closer to 1 in the observed vs. predicted plots than the MR models, implying the superiority of ANN models in prediction than MR models.

In addition, the effects of growth stage and prediction method on the errors of the prediction models were shown in Table 7. The interaction effect between growth stage and prediction method was observed (*P* < 0.05). For ADG prediction, the MAE of MR models were greater than ANN models in all growth stages (*P* < 0.01) except for 50-60 kg (*P* = 0.93), and the MAE of MR models in 60-70 kg, 80-90 kg, 90-100 kg and 100-110 kg were greater than those in 40-50 kg, 50-60 kg and 70-80 kg (*P* < 0.05). No difference was observed in different growth stages for the MAE of the ANN models. For F/G prediction, the MAE of MR models were greater than ANN models in all growth stages (*P* < 0.05) except for 70-80 kg (*P* = 0.93), and the MAE of MR models in 80-90 kg, 90-100 kg and 100-110 kg were greater than that in 50-60 kg (*P* < 0.05), while the MAE of the ANN model in 100-110 kg was greater than those in 40-50 kg and 50-60 kg (*P* < 0.05).

**Table 7:** The effect of predictive methods and growth stages on the MAE of ADG and F/G^1,2^

Item	*n*	ADG			F/G		
		MR	ANN	*P-*value	MR	ANN	*P-*value
40-50 kg	16	87^a,W^ ± 13	42^b^ ± 7	< 0.01	0.21^a,VW^ ± 0.03	0.12^b,Y^ ± 0.01	< 0.01
50-60 kg	16	79^W^ ± 11	78 ± 12	0.93	0.21^a,V^ ± 0.12	0.12^b,Y^ ± 0.05	< 0.01
60-70 kg	14	217^a,X^ ± 29	84^b^ ± 19	< 0.01	0.36^a,VWX^ ± 0.24	0.17^b^ ± 0.16	0.02
70-80 kg	12	191^a,WX^ ± 27	81^b^ ± 13	< 0.01	0.24^VW^ ± 0.22	0.25 ± 0.17	0.9
80-90 kg	9	252^a,XY^ ± 164	102^b^ ± 65	0.02	0.47^a,WXY^ ± 0.06	0.2^b^ ± 0.03	< 0.01
90-100 kg	13	306^a,XY^ ± 106	72^b^ ± 40	< 0.01	0.77^a,YZ^ ± 0.30	0.18^b^ ± 0.11	< 0.01
100-110 kg	16	364^a,YZ^ ± 117	81^b^ ± 57	< 0.01	0.91^a,Z^ ± 0.41	0.33^b,Z^ ± 0.25	< 0.01
*P-*value		< 0.01	0.15	#	< 0.01	0.01	#

*MAE* mean absolute error, *ADG* average daily gain, *F/G* feed conversion ratio.

^1^ Values are presented as means ± SEM. ^a-b^ in the same line means the MAE with different superscripts differ in predictive methods (*P* < 0.05). ^V-Z^ in the same column means the MAE with different superscripts differ in growth stages (*P* < 0.05). Pound sign means an interactive effect of methods and growth stages (*P* < 0.05).

^2^ The MAE were calculated by using predicted values and observed values in the validation data set (animal trial).

**Fig. 6:**
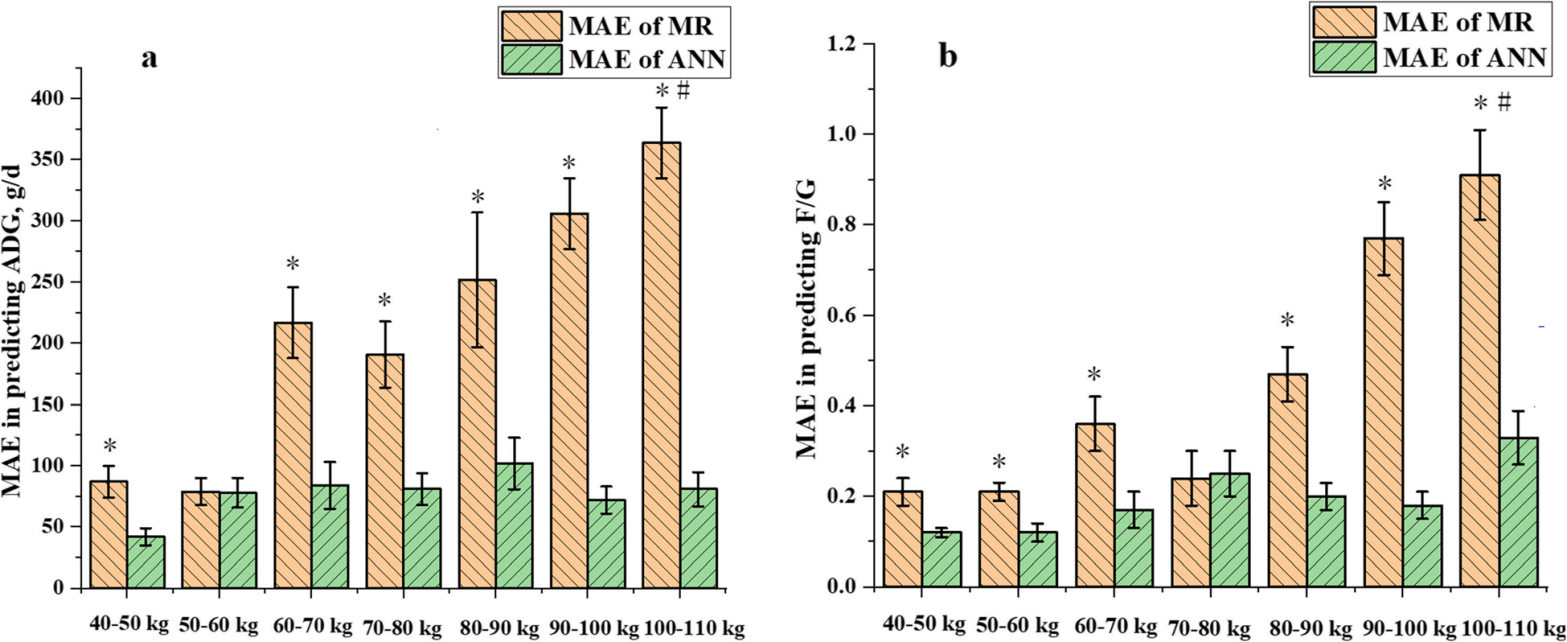
The MAE of MR and ANN models in predicting ADG (**a**) and F/G (**b**) in different growth stages. The MAE was calculated by using the predicted values and observed values in the validation data set (animal trial). * represents a significant difference between MR models and ANN models. # represents the growth stages have a significant effect on the MAE of prediction models.

Figure 6 illustrated the effect of growth stages on predictive performance of MR and ANN models in ADG and F/G prediction. The MAE of MR models exhibited increased tendency as BW increased, while the MAE of ANN models remained relatively stable. Meanwhile, ANN showed lower MAE than MR models in most growth stages (*P* < 0.05).

## Discussion

In the simulating and predictive models, determining the input variables is one of the main tasks. Inclusion of irrelevant variables not only doesn't help prediction but can reduce forecasting accuracy through added noise or systematic bias. The most sensitive variables to predict ADG or F/G selected in the current study were BW, NE intake and SID Lys intake. Body weight represents the current physiological state of pigs, which is an important factor that could determine the feed intake and nutrient digestibility of pigs [22]. As pigs grow, more feed is consumed to meet their requirements, leading to greater energy intake, which is mainly used for maintenance and then body weight gain, thus the NE intake makes a great contribution to the growth performance of growing-finishing pigs [15]. The inclusion of SID Lys intake in prediction models was in accordance with the previous reports, which concluded that SID Lys intake had a significant effect on the growth performance of pigs [26, 27]. The specific patterns of ADG influenced by SID Lys intake and NE intake were further illustrated in the current study. According to NRC (2012) [14], 100 g protein deposition in pigs requires nearly 10 g SID Lys. In the MR models built in this study, 38 g/d SID Lys intake would contribute to the highest ADG of 450 g/d, indicating greater efficiency than that reported in NRC (2012), which may be because the latter is an average value of the whole growth period. The declining trend of ADG with greater SID Lys intake more than 38 g/d could be interpreted in two aspects. On one hand, excess lysine intake would have an antagonistic action with other AAs (i.e., arginine, citrulline), which could cause the deficiency of other AAs, impair the protein accretion, and result in the retarded body growth [28]. On the other hand, the increased SID Lys intake is more likely to occur in a higher BW stage, during which period the growth performance of pigs is less affected by lysine intake [27]. As pigs grow, the increased energy requirements and more developed digestive tracts would result in greater feed intake and NE intake, among which the energy consumed beyond the maintenance requirement would deposit as protein or lipid [29]. This can interpret the positive relationship between NE intake and ADG in the current study. However, the deposition patterns for protein and lipid are different, with excess energy being used to deposit protein firstly at a cost of 10.6 kcal/g ME, and then to deposit lipid at a cost of 10.6 kcal/g ME, but the maximal rate of protein deposition (P_dmax_) was not affected by BW [29–31]. Therefore, more NE intake was deposited as fat in the later growth stages of pigs, in accordance with the decreased slope in the developed model of NE intake vs. ADG in this study as NE intake gradually increased. Even though the regression models cannot always interpret the contribution of nutrients to the growth performance of pigs precisely, the above results indicated that the MR models generated in this study were successful, and could be helpful in optimizing the feeding strategies and decisions in pig production.

The results of the current study further confirmed the previous reports that the accuracy of ANN models was influenced by their architecture. Cross et al. [32] reported that the prediction performance of ANN models relied on the number of hidden layers, the activation function, and the number of neurons in the hidden layers. Insufficient numbers of neurons could limit the capacity of ANN to learn associations between inputs and outputs, while excess numbers of neurons may lead to undesirable effects of "learning rules by memorizing" instead of learning by generalizing the acquired information, which is usually known as "over-fitting" [33, 34]. Boger and Guterman [35] stated that the number of neurons in the hidden layer of ANN models should be between 70% and 90% of the number of inputs. Blum [36] reported a general "rule of thumb" for selecting the number of neurons, which was recommended to be between the number of input and output variables. The optimal number of nodes in ADG and F/G prediction models developed in this study were 4 and 6, which is reasonable according to the above literature because the number of input and output variables in this study were 6 and 1, respectively. Furthermore, the activation function is also an imperative hyper-parameter in ANN, which can influence the accuracy of ANN by dealing with the weighting process between the hidden layer and output layer. The radial basis function was chosen in this study because it’s a powerful technique for interpolation in multidimensional space, especially suitable for modelling time series (or dynamic) relationships [37].

It’s surprising that the MR model for ADG prediction developed in the current study was found over-fitted, which did not occur for the ANN models. In many cases, MR models suffer from the prior assumption relationships between variables, thus always leading to "under-fitting" of the results [38]. Instead, the MR model generated to predict ADG in this study showed high accuracy in the training phase but failed to predict ADG with high precision in testing phase. Veum et al. [39] reported that the MR models could exhibit a high accuracy in a relatively large sample size of *n* = 496. With *n* = 287, the large sample size may attribute to the relatively high R^2^ of the MR models achieved in this study. Differing from the MR models, there is a higher risk for the phenomenon of "over-fitting" occurring in ANN models because the run mode of ANN is to obtain a local optimal solution rather than a global optimal solution [40]. The supervised learning algorithm and penalty method were applied in ANN models, which can stop the learning process when the algorithm produced a larger error in the testing data set. But this method cannot be applied in MR models, and this may explain why the “over-fitting” occurred only in MR models.

The major finding of this study was that the ANN models were more flexible and accurate than the MR models in predicting the ADG or F/G of growing-finishing pigs. These results were consistent with the previous studies that reported the precision of ANN models were better than MR methods in ruminant nutrition or edaphology [8, 10, 21]. The better performance of ANN over MR models is mainly because the conventional MR model requires an assumption regression relationship (linear or non-linear) between input variables and output variables, which greatly limits the flexibility of the prediction [41]. The existing associations between input and output variables may not follow the pre-assumption of MR models, while ANN models do not make assumptions related to data distribution, such as homoscedasticity and normality of the residual errors [42]. Moreover, the accuracy of ANN models would be improved after careful selection of the structure and hyperparameters (i.e., hidden layers, nodes and activation functions) [21]. This could also explain the outperformance of the ANN models than the MR models. Large-scaled comparisons between those two models have illustrated that the ANN models would outperform the MR models when using relatively large datasets (*n* > 20,000), while the opposite pattern occurred for small datasets [43, 44]. However, Margenot et al. [21] reported that the ANN models exhibited a better accuracy than the MR models on soil permanganate oxidizable carbon prediction in a data size of *n* = 144. As a result, the sample size in the current study (*n* = 287) was believed to be enough to predict the ADG and F/G of growing-finishing pigs using careful trained ANN models. It should be highlighted that the ANN models would also show a poor performance in some conditions when compared with the MR models, such as using a sample set with skewed distribution or introducing extra variables [34, 45]. Currently, the applications of ANN models in swine are limited to image identification, behaviour detection and disease detection. Based on the results of this study, the ANN models also exhibited great potential as an accurate predictive tool in swine nutrition. Nevertheless, suitable sample size and careful selection of the structure and hyperparameters of ANN models are required to achieve good prediction performance.

We previously found that the prediction error of MR models increased with BW increased, so we speculated that growth stages may affect the accuracy of predictive models, which was eventually proved by the results of the animal trial. Many detailed studies had revealed the effect of growth stages (or BW) on the nutrient utilization [46], organ development [47], gut microbiota [48] and biochemical indices such as enzyme activities [49] of pigs, indicating the complex physiological status in different growth stages. The MR models assumed a stable relationship (whether linear or non-linear) between the variables in whole growth period, which is a rigid assumption that may be against the dynamic real conditions. As a result, the MR models could not fully capture the highly complex relations between growth traits and other indicators [50]. Instead, ANN is more capable to mimic the dynamic patterns between variables and is more appropriate in this situation [51]. This can interpret why the ANN models were less affected by growth stages on prediction performance compared with the MR models in the current study, especially for the greater MAE of the MR models in later growth stages. Therefore, the use of MR models as a predictive tool is suggested in a small BW range, e.g., below the span of 30 kg according to the results of this study.

## Conclusion

Taken together, the accuracy of ANN models in predicting the growth performance of growing-finishing pigs was investigated in this study, and the results confirmed the hypothesis that BW, NE intake and SID Lys intake could be used as input variables to predict growth performance of pigs with high accuracy. Moreover, on testing and validation data set, ANN models revealed more flexible and accurate on ADG and F/G prediction after careful training compared with MR models. In addition, compared to MR models, ANN models were less affected by growth stages. Therefore, it is promising to use ANN models in related swine nutrition studies in the future.

## Supplementary Information

**Additional file 1: Table S1.** The information of the papers used in this study. **Table S2.** The statistic information of training data set and testing data set. **Table S3.** Ingredients and nutrient compositions of the experimental diets in the animal trial (as-fed basis). **Table S4.** The validation sample obtained by the animal trial.

## Data Availability

The data were shown in the main manuscript and supplemental materials.

## Abbreviations

ADG: Average daily gain
AIC: Akaike information criteria
ANN: Artificial neural network
BIC: Bayesian information criteria
BW: Body weight
CCC: Concordance correlation coefficients
F/G: Feed conversion ration
MAE: Mean absolute error
MR: Multiple regression
NE: Net energy
RMSE: Root mean square error
SID Lys: Standardized ileal digestible lysine
