# Correction: Predicting the growth performance of growing-finishing pigs based on net energy and digestible lysine intake using multiple regression and artificial neural networks models

**DOI:** 10.1186/s40104-022-00778-0

**Published:** 2022-10-09

**Authors:** Li Wang, Qile Hu, Lu Wang, Huangwei Shi, Changhua Lai, Shuai Zhang

**Affiliations:** State Key Laboratory of Animal Nutrition, College of Animal Science and Technology, China Agricultural University, Beijing, 100193 People’s Republic of China

**Correction: J Anim Sci Biotechnol 13, 57 (2022)**

**https://doi.org/10.1186/s40104-022-00707-1**

After publication of this article [1], it was brought to our attention that Figs. 2 and 3 were misplaced, the correct Figs. 2 and 3 are shown below:

**Fig. 2:**
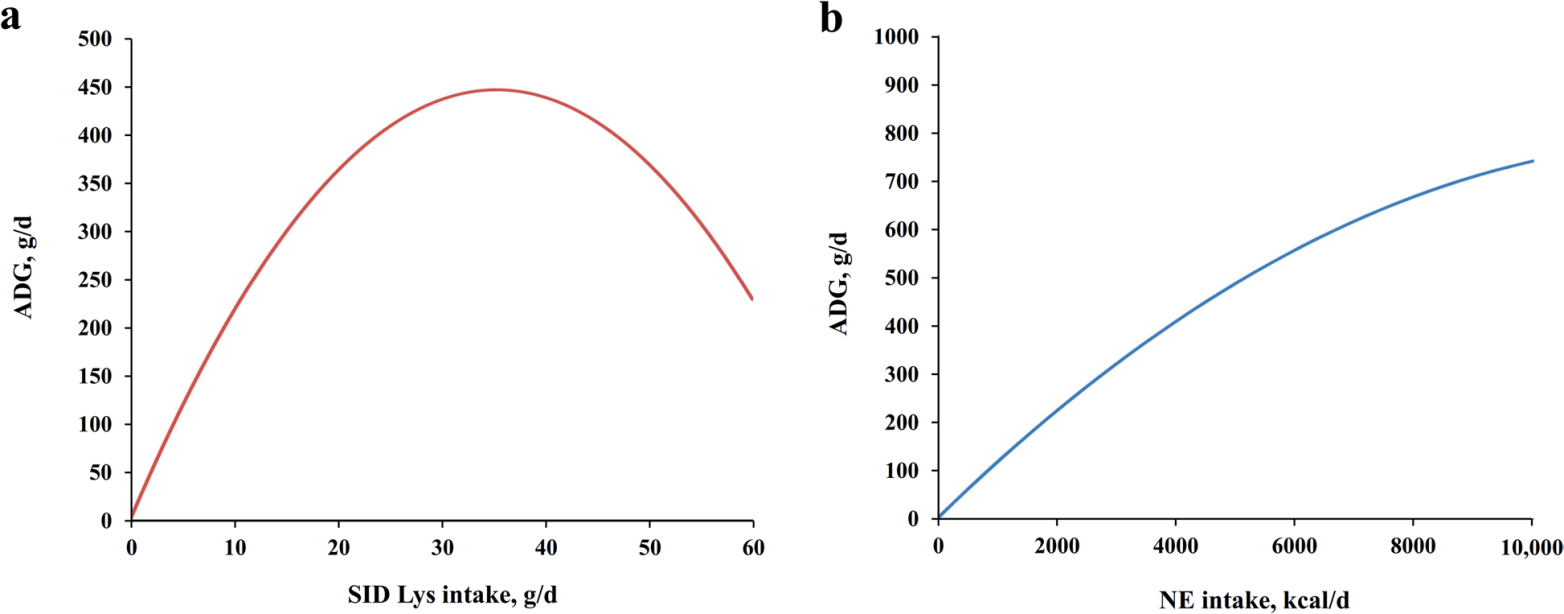
The response of ADG on different SID Lys intake (**a**) and NE intake (**b**). The curves were generated by the best fitted MR models in training. Only SID Lys intake and SID Lys intake^2^ were considered as input variables in Fig. 2a while other variables were neglected. Only NE intake and NE intake^2^ were considered as input variables in Fig. 2b

**Fig. 3:**
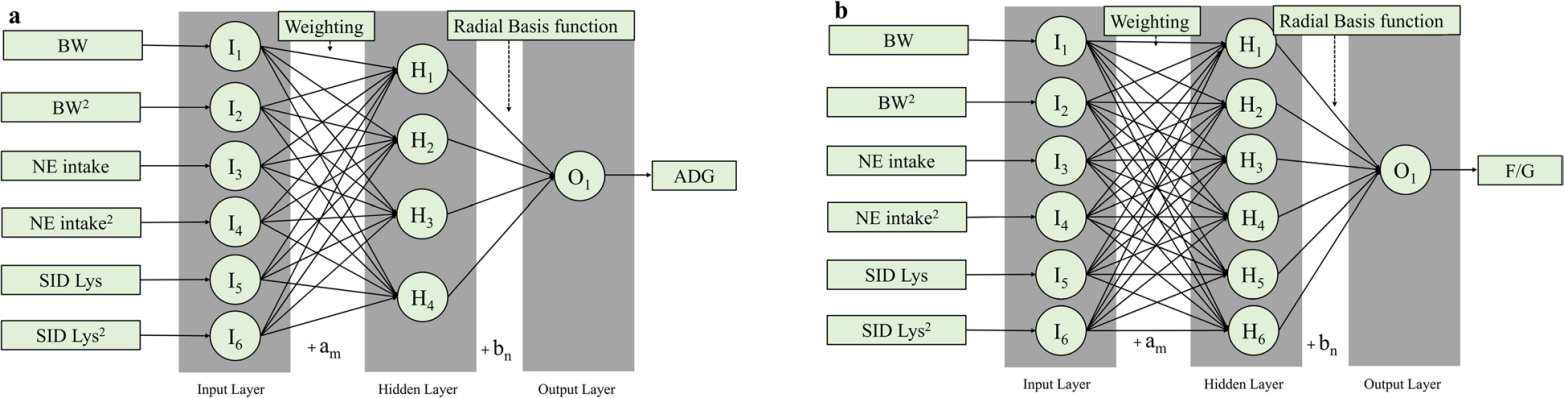
The structure of the best-fitted artificial neural networks in predicting ADG (**a**) and F/G (**b**). *H*_1_ was the value in the 1st node in the hidden layer; *I*_1_ was the 1st input; am was the bias; *O*_1_ was the value of the 1st output variable; *H*_1_ was the value of the 1st node; *b*_*n*_ was the bias; *F*_*activation*_ was the activation function

The original publication has been corrected.
