# Supplementary material for: Predicting the growth performance of growing-finishing pigs based on net energy and digestible lysine intake using multiple regression and artificial neural networks models
Source: J Anim Sci Biotechnol. 2022 May 13;13:57. doi: 10.1186/s40104-022-00707-1 (PMC9102637; doi:10.1186/s40104-022-00707-1)
Supplement: Supplementary file 1 — Additional file 1: Table S1. The information of the papers used in this study. Table S2. The statistic information of training data set and testing data set. Table S3. Ingredients and nutrient compositions of the experimental diets in the animal trial (as-fed basis). Table S4. The validation sample obtained by the animal trial. [file 40104_2022_707_MOESM1_ESM.docx]

**Table S1.** The information of the papers used in this study^1^

| Authors | Years | Journal | Breeds | Diets |
| --- | --- | --- | --- | --- |
| Yoon SY | 2010 | Journal of animal science | Lan×Y×D | Corn-SBM |
| Meng QW | 2010 | Journal of animal science | Lan×Y×D | Corn-SBM |
| Jin YH | 2010 | Journal of animal science | Lan×Y×D | Corn-SBM |
| Kim YW | 2011 | Asian-australasian journal of animal sciences | Lan×Y×D | Corn-SBM |
| Rodríguez-Sánchez JA | 2011 | Journal of animal science | Lan×Y×D | Corn-SBM |
| Newman DJ | 2011 | Journal of animal science | Lan×Y×D | Corn-SBM |
| Roux ML | 2011 | Journal of animal science | Lan×Y×D | Corn-SBM |
| Cho SB | 2012 | Asian-australasian journal of animal sciences | Lan×Y×D | Corn-SBM |
| Chu LC | 2012 | [Journal of Animal and Veterinary Advances](http://sci.justscience.cn/details.html?sci=1&id=9050) | Lan×Y×D | Corn-SBM |
| Pengfei L | 2012 | Journal of animal science and biotechnology | Lan×Y×D | Corn-SBM |
| Zhang GJ | 2012 | LivestockScience | Lan×Y×D | Corn-SBM |
| Shihai Z | 2013 | Amino Acids | Lan×Y×D | Corn-SBM |
| Zhang ZF | 2013 | Journal of Applied Animal Science | Lan×Y×D | Corn-SBM |
| Suarez-Belloch J | 2015 | Animal | Lan×Y×D | Corn-SBM |
| Zhao PY | 2015 | Animal feed science and technology | Lan×Y×D | Corn-SBM |
| Upadhaya SD | 2015 | Asian-australasian journal of animal sciences | Lan×Y×D | Corn-SBM |
| Ren M | 2015 | Asian-australasian journal of animal sciences | Lan×Y×D | Corn-SBM |
| Guo JY | 2015 | Journal of animal science | Lan×Y×D | Corn-SBM |
| Maderia MS | 2015 | Journal of animal science | Lan×Y×D | Corn-SBM |
| Morales A | 2015 | Journal of animal science | Lan×Y×D | Corn-SBM |
| Liu XT | 2015 | Journal of animal science | Lan×Y×D | Corn-SBM |
| Ma WF | 2015 | Journal of animal science | Lan×Y×D | Corn-SBM |
| Peng X | 2016 | Animal | Lan×Y×D | Corn-SBM |
| Xialan R | 2016 | Animal feed science and technology | Lan×Y×D | Corn-SBM |
| Xin W | 2016 | Animal nutrition | Lan×Y×D | Corn-SBM |
| Shihai Z | 2016 | Animal science journal | Lan×Y×D | Corn-SBM |
| Casas GA | 2016 | Journal of animal science | Lan×Y×D | Corn-SBM |
| Mejiacanos GA | 2017 | Animal feed science and technology | Lan×Y×D | Corn-SBM |
| Roselyn Kahindi | 2017 | Animal nutrition | Lan×Y×D | Corn-SBM |
| Balachandar | 2017 | Animal science journal | Lan×Y×D | Corn-SBM |
| Sung HD | 2017 | Asian-australasian journal of animal sciences | Lan×Y×D | Corn-SBM |
| Tianshui L | 2017 | [Italian Journal of Animal Science](http://sci.justscience.cn/details.html?sci=1&id=9203) | Lan×Y×D | Corn-SBM |
| Wenchao L | 2017 | [Italian Journal of Animal Science](http://sci.justscience.cn/details.html?sci=1&id=9203) | Lan×Y×D | Corn-SBM |
| Kahindi RK | 2017 | [Journal of Animal Physiology and Animal Nutrition](http://sci.justscience.cn/details.html?sci=1&id=5199) | Lan×Y×D | Corn-SBM |
| Li YH | 2017 | [Journal of Animal Physiology and Animal Nutrition](http://sci.justscience.cn/details.html?sci=1&id=5199) | Lan×Y×D | Corn-SBM |
| Espinosa SD | 2017 | Journal of animal science | Lan×Y×D | Corn-SBM |
| Jaworski NW | 2017 | Journal of animal science | Lan×Y×D | Corn-SBM |
| Velayudhan DE | 2017 | Journal of animal science | Lan×Y×D | Corn-SBM |
| Zhongchao L | 2017 | Journal of animal science and biotechnology | Lan×Y×D | Corn-SBM |
| Yu HT | 2017 | Journal of animal science and biotechnology | Lan×Y×D | Corn-SBM |
| Jinbiao Z | 2018 | Animal feed science and technology | Lan×Y×D | Corn-SBM |
| Shang QH | 2018 | Animal feed science and technology | Lan×Y×D | Corn-SBM |
| Hua Z | 2018 | Animal science journal | Lan×Y×D | Corn-SBM |
| Bing D | 2018 | Asian-australasian journal of animal sciences | Lan×Y×D | Corn-SBM |
| Yetong X | 2018 | Asian-australasian journal of animal sciences | Lan×Y×D | Corn-SBM |
| Xiaoya Z | 2018 | [Journal of Agricultural and Food Chemistry](http://sci.justscience.cn/details.html?sci=1&id=1652) | Lan×Y×D | Corn-SBM |
| Lee | 2018 | Journal of animal science | Lan×Y×D | Corn-SBM |
| He DT | 2018 | Journal of animal science | Lan×Y×D | Corn-SBM |
| Fei Y | 2018 | Journal of animal science | Lan×Y×D | Corn-SBM |
| Hongbo Y | 2018 | Journal of animal science | Lan×Y×D | Corn-SBM |
| Yanhong L | 2018 | Journal of animal science and biotechnology | Lan×Y×D | Corn-SBM |
| Chusak P | 2018 | Journal of Applied Animal Science | Lan×Y×D | Corn-SBM |
| Xun P | 2018 | [Journal of the Science of Food and Agriculture](http://sci.justscience.cn/details.html?sci=1&id=4400) | Lan×Y×D | Corn-SBM |
| Huan S | 2019 | Animal feed science and technology | Lan×Y×D | Corn-SBM |
| Chunchun W | 2019 | Animal feed science and technology | Lan×Y×D | Corn-SBM |
| Wang YM | 2019 | Animal feed science and technology | Lan×Y×D | Corn-SBM |
| Xiaokang M | 2019 | Animal feed science and technology | Lan×Y×D | Corn-SBM |
| Hongnan L | 2019 | Animal nutrition | Lan×Y×D | Corn-SBM |
| Xinjian L | 2019 | Animal science journal | Lan×Y×D | Corn-SBM |
| Hua Z | 2019 | Animal science journal | Lan×Y×D | Corn-SBM |
| Hufang L | 2019 | Asian-australasian journal of animal sciences | Lan×Y×D | Corn-SBM |
| Shaoshuai L | 2019 | Asian-australasian journal of animal sciences | Lan×Y×D | Corn-SBM |
| Liu JB | 2019 | Asian-australasian journal of animal sciences | Lan×Y×D | Corn-SBM |
| Nguyen DH | 2019 | [Canadian Journal of Animal Science](http://sci.justscience.cn/details.html?sci=1&id=5894) | Lan×Y×D | Corn-SBM |
| Jinbiao Z | 2019 | Food and Agricultural Immunology | Lan×Y×D | Corn-SBM |
| Jonathan N | 2019 | Journal of animal science | Lan×Y×D | Corn-SBM |
| Jianying Z | 2019 | Journal of animal science | Lan×Y×D | Corn-SBM |
| Xindi Y | 2019 | Journal of animal science | Lan×Y×D | Corn-SBM |
| Linhu F | 2019 | [Journal of Animal Science and Technology](http://sci.justscience.cn/details.html?sci=1&id=13317) | Lan×Y×D | Corn-SBM |
| Jiwen Y | 2019 | Journal of Integrative Agriculture | Lan×Y×D | Corn-SBM |
| Ruixia L | 2019 | [Journal of the Science of Food and Agriculture](http://sci.justscience.cn/details.html?sci=1&id=4400) | Lan×Y×D | Corn-SBM |
| Jun C | 2019 | Journal of Trace Elements in Medicine and Biology | Lan×Y×D | Corn-SBM |

^1^Totally, 72 papers from 2010-2019 were selected for further analysis. The selection criteria were described in the previous part.

**Table S2.** The statistic information of training data set and testing data set^1^

| ADG, g/d | ADFI, g/d | F/G | BW, kg | NE intake, kcal/d | SID Lys intake, g/d | NDF intake g,d | ADF intake, g/d | CP intake g/d | SID Met intake, g/d | SID Thr intake, g/d | SID trp intake, g/d | SID val intake, g/d | data set |
| --- | --- | --- | --- | --- | --- | --- | --- | --- | --- | --- | --- | --- | --- |
| 202 | 264 | 1.31 | 5.50 | 690.99 | 3.79 | 25.83 | 7.09 | 52.51 | 2.21 | 2.95 | 0.48 | 2.23 | training |
| 207 | 261 | 1.26 | 5.51 | 676.23 | 4.29 | 15.32 | 4.35 | 55.95 | 1.17 | 2.18 | 0.66 | 2.58 | training |
| 207 | 263 | 1.27 | 5.51 | 710.12 | 3.77 | 15.53 | 4.72 | 61.02 | 1.39 | 2.38 | 0.72 | 2.80 | training |
| 233 | 289 | 1.24 | 6.59 | 778.71 | 4.13 | 36.27 | 11.74 | 40.01 | 0.68 | 1.13 | 0.27 | 1.58 | training |
| 237 | 291 | 1.23 | 6.60 | 781.36 | 4.54 | 16.43 | 4.83 | 63.54 | 1.67 | 2.48 | 0.75 | 2.93 | training |
| 243 | 293 | 1.21 | 6.62 | 784.18 | 4.93 | 16.49 | 4.91 | 64.90 | 1.98 | 2.54 | 0.77 | 2.99 | training |
| 244 | 295 | 1.21 | 6.62 | 786.88 | 5.32 | 16.52 | 4.99 | 66.18 | 2.38 | 2.73 | 0.79 | 3.04 | training |
| 237 | 322 | 1.36 | 7.07 | 871.50 | 4.67 | 24.07 | 7.02 | 72.96 | 1.63 | 2.79 | 0.82 | 3.29 | training |
| 252 | 341 | 1.35 | 7.12 | 882.55 | 5.57 | 25.20 | 6.71 | 67.49 | 1.53 | 2.58 | 0.75 | 3.07 | training |
| 260 | 363 | 1.40 | 8.10 | 917.24 | 5.10 | 35.07 | 9.88 | 63.91 | 2.53 | 2.55 | 1.18 | 3.05 | training |
| 266 | 349 | 1.33 | 8.15 | 882.43 | 4.91 | 36.94 | 5.89 | 81.83 | 2.00 | 2.77 | 0.87 | 3.56 | training |
| 205 | 328 | 1.61 | 8.30 | 823.81 | 4.51 | 35.90 | 24.62 | 66.83 | 1.84 | 2.08 | 0.64 | 2.68 | training |
| 213 | 316 | 1.50 | 8.35 | 773.35 | 4.40 | 30.71 | 12.38 | 66.95 | 1.82 | 2.32 | 0.65 | 2.82 | training |
| 209 | 334 | 1.59 | 8.35 | 795.92 | 4.71 | 31.98 | 20.19 | 70.64 | 1.95 | 2.38 | 0.71 | 2.93 | training |
| 252 | 424 | 1.68 | 8.40 | 1064.86 | 5.83 | 64.59 | 38.02 | 97.92 | 1.93 | 2.93 | 0.81 | 3.85 | training |
| 279 | 372 | 1.33 | 8.45 | 991.73 | 5.66 | 28.39 | 9.04 | 76.49 | 2.49 | 3.01 | 0.79 | 3.32 | training |
| 258 | 428 | 1.66 | 8.45 | 1047.44 | 5.96 | 41.91 | 12.23 | 90.76 | 2.42 | 3.19 | 0.88 | 3.86 | training |
| 258 | 423 | 1.64 | 8.45 | 1057.90 | 5.96 | 40.15 | 20.95 | 89.25 | 2.43 | 3.05 | 0.89 | 3.73 | training |
| 261 | 466 | 1.78 | 8.45 | 1110.41 | 6.56 | 44.96 | 23.22 | 98.64 | 2.69 | 3.37 | 0.98 | 4.12 | training |
| 281 | 378 | 1.35 | 8.46 | 1004.38 | 6.20 | 28.68 | 9.25 | 78.76 | 2.95 | 3.71 | 0.81 | 3.42 | training |
| 238 | 300 | 1.26 | 8.49 | 734.82 | 4.63 | 28.68 | 4.39 | 51.32 | 2.19 | 2.08 | 0.57 | 2.65 | training |
| 239 | 369 | 1.54 | 8.76 | 964.59 | 3.30 | 34.97 | 6.00 | 72.48 | 2.26 | 2.82 | 0.81 | 3.28 | training |
| 142 | 290 | 2.12 | 8.76 | 733.43 | 3.82 | 37.24 | 12.76 | 60.72 | 1.26 | 2.02 | 0.57 | 2.52 | training |
| 142 | 259 | 1.83 | 8.90 | 601.70 | 3.61 | 38.96 | 16.71 | 58.88 | 1.18 | 1.83 | 0.50 | 2.32 | training |
| 231 | 386 | 1.67 | 8.93 | 1002.28 | 5.80 | 53.18 | 19.27 | 46.16 | 1.54 | 1.42 | 0.63 | 1.73 | training |
| 330 | 451 | 1.37 | 8.96 | 1153.16 | 5.67 | 50.62 | 12.34 | 58.46 | 0.99 | 1.76 | 0.44 | 2.40 | training |
| 155 | 293 | 1.89 | 8.97 | 740.50 | 3.88 | 36.53 | 11.98 | 61.40 | 1.27 | 2.06 | 0.59 | 2.56 | training |
| 334 | 429 | 1.28 | 8.97 | 1154.81 | 5.99 | 38.18 | 11.22 | 93.42 | 3.94 | 3.61 | 1.60 | 4.01 | training |
| 237 | 380 | 1.60 | 8.98 | 989.94 | 3.37 | 36.30 | 5.58 | 65.30 | 2.74 | 2.65 | 0.72 | 2.99 | training |
| 163 | 309 | 1.89 | 8.98 | 780.33 | 4.15 | 37.38 | 11.67 | 64.83 | 1.33 | 2.20 | 0.62 | 2.72 | training |
| 266 | 379 | 1.42 | 9.07 | 1014.35 | 4.02 | 36.14 | 9.45 | 77.96 | 1.68 | 2.79 | 0.80 | 3.47 | training |
| 347 | 443 | 1.28 | 9.07 | 1184.30 | 6.63 | 39.56 | 10.60 | 81.75 | 3.09 | 3.11 | 1.22 | 3.52 | training |
| 199 | 322 | 1.62 | 9.15 | 748.96 | 5.04 | 43.73 | 19.28 | 70.61 | 1.80 | 2.28 | 0.63 | 2.83 | training |
| 178 | 314 | 1.78 | 9.16 | 792.32 | 4.24 | 36.81 | 10.88 | 65.97 | 1.31 | 2.26 | 0.64 | 2.79 | training |
| 367 | 432 | 1.18 | 9.17 | 1132.89 | 7.42 | 38.69 | 11.35 | 94.25 | 3.41 | 3.55 | 1.44 | 4.04 | training |
| 368 | 452 | 1.23 | 9.20 | 1191.59 | 7.50 | 41.00 | 11.99 | 98.98 | 2.40 | 3.59 | 1.15 | 4.25 | training |
| 379 | 439 | 1.16 | 9.22 | 1157.59 | 7.47 | 39.55 | 11.49 | 94.57 | 3.50 | 3.56 | 1.41 | 4.06 | training |
| 362 | 476 | 1.32 | 9.24 | 1215.37 | 6.60 | 36.79 | 11.59 | 104.42 | 4.72 | 4.04 | 2.20 | 4.73 | training |
| 381 | 450 | 1.18 | 9.24 | 1186.32 | 7.20 | 40.31 | 11.83 | 98.17 | 4.59 | 4.69 | 1.86 | 4.21 | training |
| 374 | 486 | 1.30 | 9.29 | 1239.83 | 6.74 | 37.52 | 11.82 | 106.59 | 4.82 | 4.12 | 2.24 | 4.82 | training |
| 395 | 464 | 1.18 | 9.41 | 1179.73 | 8.24 | 35.66 | 11.25 | 101.65 | 4.60 | 3.93 | 2.14 | 4.60 | training |
| 401 | 499 | 1.25 | 9.47 | 1272.30 | 7.57 | 38.49 | 12.13 | 109.42 | 4.95 | 4.23 | 2.30 | 4.95 | training |
| 385 | 485 | 1.25 | 9.49 | 1233.79 | 7.98 | 37.30 | 11.77 | 106.27 | 4.81 | 4.11 | 2.24 | 4.81 | training |
| 409 | 477 | 1.16 | 9.52 | 1211.72 | 8.47 | 36.61 | 11.56 | 104.46 | 4.73 | 4.04 | 2.20 | 4.73 | training |
| 389 | 683 | 1.76 | 9.55 | 1809.84 | 8.86 | 66.07 | 21.82 | 134.29 | 2.62 | 4.90 | 1.48 | 5.48 | training |
| 419 | 521 | 1.27 | 9.63 | 1301.93 | 8.60 | 72.46 | 19.53 | 77.90 | 1.38 | 2.87 | 0.71 | 3.43 | training |
| 421 | 523 | 1.19 | 9.65 | 1294.71 | 8.51 | 45.45 | 13.24 | 119.86 | 3.03 | 4.44 | 1.26 | 5.27 | training |
| 267 | 389 | 1.47 | 9.65 | 989.11 | 5.82 | 42.56 | 10.33 | 48.42 | 1.10 | 1.49 | 0.36 | 1.99 | training |
| 389 | 632 | 1.62 | 9.89 | 1687.28 | 8.21 | 58.11 | 18.66 | 128.78 | 2.50 | 4.59 | 1.41 | 5.35 | training |
| 279 | 473 | 1.25 | 9.90 | 1301.63 | 6.18 | 26.69 | 7.96 | 92.59 | 2.25 | 3.32 | 0.91 | 3.85 | training |
| 286 | 383 | 1.34 | 9.92 | 1004.01 | 5.32 | 33.42 | 8.44 | 61.05 | 1.91 | 3.18 | 0.98 | 3.66 | training |
| 403 | 675 | 1.67 | 9.94 | 1815.15 | 8.79 | 58.81 | 18.28 | 142.36 | 2.76 | 5.09 | 1.54 | 6.00 | training |
| 286 | 383 | 1.34 | 9.97 | 1002.67 | 5.50 | 33.25 | 8.64 | 60.51 | 1.84 | 3.43 | 1.06 | 3.91 | training |
| 303 | 366 | 1.21 | 10.09 | 942.43 | 5.19 | 33.20 | 9.96 | 73.95 | 1.44 | 3.19 | 0.94 | 3.18 | training |
| 303 | 366 | 1.21 | 10.15 | 945.99 | 5.02 | 33.15 | 9.45 | 75.53 | 1.49 | 2.96 | 0.88 | 3.24 | training |
| 303 | 366 | 1.21 | 10.15 | 945.87 | 5.30 | 34.32 | 9.64 | 64.55 | 2.65 | 2.31 | 1.89 | 2.63 | training |
| 303 | 366 | 1.21 | 10.15 | 945.97 | 5.30 | 34.32 | 9.64 | 64.55 | 2.65 | 2.31 | 1.89 | 2.63 | training |
| 355 | 456 | 1.28 | 10.44 | 1216.74 | 5.98 | 44.76 | 14.87 | 88.84 | 1.60 | 2.91 | 0.83 | 3.64 | training |
| 170 | 335 | 1.97 | 10.45 | 843.72 | 4.54 | 44.84 | 12.62 | 66.59 | 1.62 | 2.29 | 0.67 | 2.87 | training |
| 365 | 583 | 1.60 | 10.56 | 1510.57 | 8.79 | 42.29 | 12.49 | 132.12 | 2.94 | 5.06 | 1.49 | 5.96 | training |
| 380 | 592 | 1.56 | 10.88 | 1582.85 | 8.91 | 57.76 | 14.97 | 121.75 | 2.57 | 4.35 | 1.24 | 5.42 | training |
| 391 | 628 | 1.61 | 10.92 | 1580.51 | 7.83 | 52.70 | 16.17 | 153.25 | 8.96 | 5.55 | 1.52 | 6.61 | training |
| 392 | 621 | 1.58 | 10.94 | 1526.32 | 9.06 | 62.13 | 16.43 | 129.93 | 5.54 | 4.94 | 2.36 | 5.80 | training |
| 397 | 626 | 1.57 | 10.95 | 1560.69 | 7.95 | 55.67 | 16.63 | 136.49 | 8.56 | 4.84 | 1.32 | 5.83 | training |
| 222 | 391 | 1.76 | 11.00 | 990.31 | 5.47 | 53.57 | 15.54 | 86.72 | 1.56 | 3.00 | 0.88 | 3.73 | training |
| 379 | 563 | 1.55 | 11.09 | 1502.30 | 8.72 | 67.46 | 24.14 | 73.88 | 1.27 | 2.24 | 0.60 | 3.11 | training |
| 234 | 433 | 1.85 | 11.13 | 1056.74 | 6.03 | 75.72 | 21.33 | 63.69 | 0.92 | 1.83 | 0.54 | 2.61 | training |
| 421 | 550 | 1.34 | 11.15 | 1460.00 | 7.42 | 53.38 | 11.27 | 96.61 | 5.76 | 3.65 | 3.02 | 5.32 | training |
| 350 | 536 | 1.53 | 11.25 | 1365.97 | 7.08 | 53.50 | 20.24 | 108.81 | 2.30 | 4.23 | 1.24 | 5.10 | training |
| 441 | 560 | 1.35 | 11.30 | 1481.09 | 8.26 | 56.58 | 14.65 | 113.39 | 3.37 | 4.01 | 1.28 | 4.77 | training |
| 362 | 655 | 1.79 | 11.30 | 1710.52 | 8.74 | 57.42 | 17.38 | 140.26 | 2.46 | 5.13 | 1.48 | 6.19 | training |
| 358 | 549 | 1.53 | 11.31 | 1428.15 | 7.25 | 53.33 | 18.86 | 110.31 | 2.31 | 4.25 | 1.25 | 5.02 | training |
| 362 | 551 | 1.52 | 11.33 | 1462.63 | 7.24 | 52.06 | 17.05 | 109.58 | 2.26 | 4.18 | 1.24 | 4.84 | training |
| 429 | 543 | 1.25 | 11.42 | 1430.84 | 8.69 | 54.68 | 14.26 | 111.17 | 3.79 | 4.03 | 1.52 | 4.78 | training |
| 379 | 568 | 1.50 | 11.45 | 1537.82 | 7.46 | 52.15 | 15.63 | 111.78 | 2.28 | 4.22 | 1.27 | 4.78 | training |
| 371 | 627 | 1.69 | 11.88 | 1684.13 | 7.09 | 47.27 | 15.45 | 132.20 | 5.66 | 7.22 | 1.37 | 5.74 | training |
| 286 | 383 | 1.34 | 11.97 | 1002.65 | 6.19 | 34.74 | 10.43 | 77.38 | 2.16 | 2.92 | 0.76 | 3.32 | training |
| 386 | 624 | 1.62 | 12.04 | 1670.65 | 7.81 | 61.83 | 16.51 | 118.66 | 3.06 | 4.31 | 1.06 | 5.04 | training |
| 348 | 636 | 1.83 | 12.13 | 1559.78 | 7.71 | 74.72 | 26.31 | 118.70 | 2.41 | 5.08 | 1.65 | 4.80 | training |
| 394 | 621 | 1.58 | 12.20 | 1651.41 | 9.33 | 61.07 | 16.61 | 121.78 | 4.45 | 5.80 | 1.10 | 5.17 | training |
| 303 | 366 | 1.21 | 12.21 | 942.40 | 5.53 | 39.94 | 11.59 | 61.60 | 1.56 | 2.02 | 0.54 | 2.56 | training |
| 487 | 720 | 1.47 | 12.23 | 1899.08 | 9.34 | 80.58 | 20.42 | 142.06 | 2.91 | 5.04 | 1.34 | 5.94 | training |
| 487 | 758 | 1.56 | 12.44 | 2000.55 | 9.83 | 80.03 | 27.08 | 155.66 | 11.78 | 5.23 | 1.42 | 6.54 | training |
| 382 | 633 | 1.66 | 12.61 | 1625.62 | 7.71 | 72.38 | 25.94 | 118.08 | 2.39 | 5.07 | 1.65 | 4.76 | training |
| 521 | 744 | 1.43 | 12.65 | 1958.97 | 9.65 | 83.27 | 21.10 | 146.80 | 4.05 | 5.21 | 1.38 | 6.13 | training |
| 391 | 683 | 1.75 | 12.69 | 1766.85 | 10.37 | 66.34 | 18.00 | 109.42 | 7.36 | 4.86 | 3.19 | 6.28 | training |
| 358 | 591 | 1.65 | 12.87 | 1564.08 | 7.80 | 61.77 | 13.92 | 105.41 | 2.52 | 3.64 | 1.05 | 4.52 | training |
| 416 | 788 | 1.89 | 12.91 | 2011.09 | 9.11 | 75.43 | 22.13 | 142.60 | 2.79 | 5.57 | 1.34 | 5.96 | training |
| 404 | 606 | 1.50 | 12.96 | 1586.08 | 8.19 | 70.86 | 23.57 | 112.60 | 3.81 | 3.74 | 1.03 | 4.65 | training |
| 441 | 575 | 1.30 | 13.47 | 1550.27 | 7.75 | 52.86 | 14.23 | 110.80 | 6.04 | 3.85 | 1.05 | 5.05 | training |
| 405 | 630 | 1.56 | 13.75 | 1674.86 | 6.82 | 61.93 | 18.41 | 119.24 | 3.97 | 4.61 | 1.27 | 5.18 | training |
| 477 | 847 | 1.79 | 13.88 | 2203.40 | 11.58 | 85.26 | 19.70 | 161.80 | 3.77 | 5.71 | 1.68 | 7.02 | training |
| 387 | 683 | 1.76 | 14.05 | 1536.15 | 9.69 | 116.77 | 55.47 | 162.17 | 3.23 | 4.87 | 1.33 | 6.22 | training |
| 430 | 631 | 1.47 | 14.15 | 1668.18 | 7.85 | 62.45 | 17.92 | 113.17 | 2.39 | 4.12 | 1.13 | 4.92 | training |
| 431 | 601 | 1.39 | 14.15 | 1591.31 | 8.31 | 64.46 | 31.69 | 53.76 | 2.66 | 1.75 | 1.07 | 2.72 | training |
| 383 | 607 | 1.58 | 14.15 | 1607.14 | 7.32 | 57.30 | 16.08 | 107.33 | 3.00 | 4.75 | 1.43 | 4.37 | training |
| 440 | 668 | 1.52 | 14.25 | 1731.56 | 8.65 | 61.76 | 18.60 | 120.62 | 4.04 | 5.74 | 1.73 | 6.44 | training |
| 397 | 629 | 1.58 | 14.35 | 1664.47 | 7.58 | 58.98 | 16.57 | 110.94 | 3.11 | 4.91 | 1.48 | 6.78 | training |
| 448 | 619 | 1.38 | 14.35 | 1601.96 | 9.74 | 60.84 | 17.13 | 106.89 | 4.40 | 4.26 | 1.62 | 4.84 | training |
| 458 | 636 | 1.39 | 14.50 | 1616.64 | 11.10 | 62.20 | 17.25 | 106.49 | 5.63 | 4.50 | 2.33 | 5.47 | training |
| 412 | 640 | 1.55 | 14.56 | 1657.47 | 8.41 | 57.75 | 15.81 | 123.22 | 6.71 | 4.28 | 1.17 | 5.53 | training |
| 412 | 640 | 1.55 | 14.56 | 1657.47 | 8.41 | 62.17 | 16.87 | 102.53 | 6.89 | 4.55 | 2.99 | 5.88 | training |
| 540 | 861 | 1.59 | 14.60 | 2056.34 | 11.78 | 95.47 | 46.32 | 175.82 | 4.75 | 5.66 | 1.63 | 7.17 | training |
| 390 | 760 | 1.92 | 14.65 | 1979.03 | 8.50 | 94.97 | 43.23 | 174.96 | 4.49 | 6.15 | 1.71 | 7.26 | training |
| 476 | 647 | 1.36 | 14.75 | 1695.28 | 9.11 | 63.85 | 18.18 | 114.23 | 3.53 | 4.35 | 1.14 | 4.97 | training |
| 476 | 647 | 1.36 | 14.75 | 1695.28 | 9.11 | 63.85 | 18.18 | 114.23 | 3.53 | 4.35 | 1.14 | 4.97 | training |
| 426 | 657 | 1.54 | 14.75 | 1743.54 | 7.92 | 61.62 | 17.31 | 115.89 | 3.24 | 5.13 | 1.55 | 6.29 | training |
| 426 | 657 | 1.54 | 14.75 | 1702.11 | 8.63 | 61.78 | 17.35 | 116.00 | 4.76 | 4.15 | 1.81 | 4.72 | training |
| 426 | 657 | 1.54 | 14.75 | 1702.11 | 8.63 | 61.78 | 17.35 | 116.00 | 4.76 | 4.15 | 1.81 | 4.72 | training |
| 569 | 863 | 1.52 | 14.80 | 2116.49 | 11.66 | 96.94 | 28.09 | 176.55 | 4.66 | 5.86 | 1.60 | 7.34 | training |
| 576 | 878 | 1.52 | 14.80 | 2209.87 | 11.72 | 80.68 | 61.87 | 184.31 | 5.13 | 6.13 | 1.88 | 7.57 | training |
| 569 | 899 | 1.58 | 14.85 | 2253.27 | 12.30 | 98.27 | 48.09 | 182.96 | 4.94 | 5.90 | 1.70 | 7.47 | training |
| 581 | 874 | 1.50 | 15.10 | 2195.08 | 11.81 | 95.65 | 65.60 | 178.16 | 4.92 | 5.55 | 1.70 | 7.13 | training |
| 449 | 787 | 1.75 | 15.15 | 1987.73 | 8.34 | 81.38 | 23.23 | 149.86 | 2.38 | 5.20 | 1.56 | 6.27 | training |
| 543 | 860 | 1.58 | 15.55 | 2053.95 | 11.76 | 94.73 | 55.41 | 175.46 | 4.77 | 5.55 | 1.65 | 7.09 | training |
| 585 | 862 | 1.47 | 15.95 | 2141.52 | 11.57 | 96.83 | 28.05 | 176.35 | 4.66 | 5.86 | 1.59 | 7.33 | training |
| 541 | 1047 | 1.90 | 16.28 | 2674.39 | 11.61 | 136.90 | 42.99 | 204.53 | 3.27 | 6.69 | 1.87 | 8.50 | training |
| 507 | 772 | 1.51 | 16.47 | 2049.31 | 9.72 | 83.79 | 21.55 | 121.54 | 1.99 | 3.70 | 0.89 | 4.93 | training |
| 565 | 1181 | 2.09 | 16.97 | 3011.45 | 13.39 | 145.61 | 41.13 | 231.31 | 3.51 | 7.72 | 2.16 | 9.75 | training |
| 460 | 802 | 1.74 | 17.03 | 2036.99 | 9.92 | 83.58 | 30.23 | 156.13 | 3.09 | 6.09 | 1.71 | 6.83 | training |
| 472 | 816 | 1.73 | 17.17 | 2115.36 | 10.10 | 82.84 | 27.96 | 157.08 | 3.15 | 6.15 | 1.72 | 6.65 | training |
| 551 | 961 | 1.75 | 17.40 | 2303.42 | 14.84 | 91.11 | 26.04 | 199.50 | 4.67 | 7.00 | 1.92 | 8.52 | training |
| 474 | 817 | 1.72 | 17.42 | 2161.00 | 10.05 | 80.74 | 25.20 | 155.54 | 3.24 | 6.03 | 1.70 | 6.36 | training |
| 547 | 892 | 1.63 | 17.65 | 2130.46 | 12.20 | 98.26 | 57.47 | 181.98 | 4.94 | 5.76 | 1.71 | 7.36 | training |
| 573 | 933 | 1.63 | 18.10 | 2348.30 | 12.45 | 102.11 | 70.03 | 190.18 | 5.25 | 5.92 | 1.81 | 7.62 | training |
| 571 | 938 | 1.64 | 18.15 | 2217.41 | 12.86 | 145.59 | 44.24 | 217.36 | 4.06 | 6.90 | 1.70 | 8.83 | training |
| 559 | 940 | 1.77 | 18.20 | 2161.86 | 13.04 | 144.54 | 64.31 | 217.47 | 4.17 | 6.70 | 1.75 | 8.69 | training |
| 572 | 944 | 1.65 | 18.30 | 2254.57 | 12.91 | 106.04 | 30.72 | 193.13 | 5.10 | 6.41 | 1.74 | 8.02 | training |
| 587 | 938 | 1.59 | 18.30 | 2277.86 | 12.71 | 102.66 | 70.41 | 191.20 | 5.28 | 5.95 | 1.82 | 7.66 | training |
| 553 | 853 | 1.54 | 18.73 | 2258.47 | 11.39 | 47.46 | 14.40 | 166.97 | 4.03 | 5.97 | 1.63 | 6.94 | training |
| 608 | 859 | 1.41 | 21.41 | 2153.84 | 8.71 | 91.12 | 35.71 | 194.73 | 11.80 | 6.59 | 1.83 | 8.16 | training |
| 631 | 866 | 1.37 | 21.68 | 2132.81 | 10.63 | 75.94 | 23.00 | 191.33 | 16.01 | 6.80 | 1.87 | 8.17 | training |
| 713 | 1137 | 1.59 | 23.89 | 2906.69 | 13.80 | 115.48 | 29.27 | 167.48 | 6.13 | 9.06 | 2.48 | 9.43 | training |
| 870 | 1700 | 1.96 | 32.08 | 4516.97 | 16.02 | 184.98 | 75.58 | 260.39 | 5.21 | 7.96 | 2.20 | 10.57 | training |
| 645 | 1836 | 2.85 | 32.53 | 4608.14 | 16.67 | 224.29 | 58.80 | 287.32 | 4.41 | 9.79 | 2.66 | 12.00 | training |
| 665 | 1664 | 2.50 | 32.91 | 4297.87 | 15.82 | 189.83 | 52.21 | 265.39 | 4.08 | 9.28 | 2.37 | 11.05 | training |
| 761 | 1798 | 2.36 | 34.15 | 4644.40 | 17.05 | 205.05 | 56.34 | 286.09 | 4.40 | 10.00 | 2.55 | 11.91 | training |
| 730 | 1576 | 2.16 | 35.85 | 3852.14 | 15.88 | 189.41 | 63.72 | 267.61 | 5.16 | 10.06 | 2.53 | 12.21 | training |
| 730 | 1576 | 2.16 | 36.18 | 3852.14 | 15.88 | 189.41 | 63.72 | 267.61 | 5.16 | 10.06 | 2.53 | 12.21 | training |
| 738 | 1546 | 2.09 | 36.32 | 3983.64 | 15.50 | 175.45 | 49.25 | 253.90 | 4.85 | 9.33 | 2.52 | 10.55 | training |
| 712 | 1798 | 2.53 | 36.37 | 4635.73 | 16.54 | 210.56 | 50.70 | 217.92 | 6.34 | 9.87 | 2.65 | 10.10 | training |
| 729 | 1686 | 2.31 | 36.61 | 4342.30 | 15.51 | 197.25 | 47.50 | 204.21 | 5.94 | 9.25 | 2.49 | 11.15 | training |
| 567 | 1462 | 2.56 | 36.92 | 3745.59 | 11.81 | 157.41 | 47.12 | 308.76 | 4.14 | 10.37 | 2.85 | 12.84 | training |
| 759 | 1831 | 2.41 | 37.03 | 4718.28 | 16.84 | 214.32 | 51.60 | 221.85 | 6.45 | 10.05 | 2.70 | 11.20 | training |
| 763 | 1853 | 2.43 | 37.08 | 4769.84 | 17.04 | 216.68 | 52.18 | 224.37 | 6.53 | 10.17 | 2.73 | 13.18 | training |
| 789 | 2316 | 2.94 | 37.21 | 6014.12 | 20.09 | 263.39 | 71.64 | 356.42 | 6.30 | 12.13 | 3.26 | 14.78 | training |
| 597 | 1390 | 2.30 | 37.22 | 3692.05 | 11.18 | 169.00 | 63.12 | 223.83 | 3.68 | 7.10 | 1.96 | 9.43 | training |
| 688 | 1467 | 2.13 | 37.68 | 3759.93 | 13.15 | 174.69 | 42.16 | 183.20 | 5.22 | 8.40 | 2.26 | 9.22 | training |
| 697 | 1545 | 2.22 | 37.85 | 3957.97 | 13.85 | 183.81 | 44.37 | 192.83 | 5.49 | 8.85 | 3.16 | 9.70 | training |
| 760 | 1450 | 1.91 | 37.91 | 3648.56 | 13.79 | 162.56 | 79.84 | 286.82 | 4.18 | 9.15 | 2.69 | 11.73 | training |
| 770 | 1470 | 1.89 | 38.01 | 3684.23 | 14.58 | 180.39 | 49.74 | 300.89 | 4.08 | 9.96 | 2.88 | 12.57 | training |
| 790 | 1570 | 2.00 | 38.49 | 4049.57 | 15.52 | 181.99 | 51.52 | 318.10 | 4.34 | 10.55 | 2.95 | 13.25 | training |
| 790 | 1570 | 1.99 | 38.49 | 4019.75 | 15.06 | 175.96 | 74.18 | 304.46 | 4.48 | 9.80 | 2.82 | 12.53 | training |
| 790 | 1560 | 1.96 | 38.63 | 4085.08 | 15.36 | 176.73 | 50.49 | 313.99 | 4.29 | 10.42 | 2.88 | 13.06 | training |
| 790 | 1560 | 1.97 | 38.63 | 4066.20 | 14.88 | 174.08 | 67.49 | 302.91 | 4.46 | 9.82 | 2.79 | 12.51 | training |
| 692 | 1573 | 2.27 | 38.64 | 4222.60 | 17.24 | 162.86 | 40.68 | 211.70 | 4.17 | 6.51 | 1.67 | 8.69 | training |
| 800 | 1550 | 1.92 | 38.75 | 3850.90 | 15.56 | 200.32 | 53.98 | 321.82 | 4.32 | 10.67 | 3.20 | 13.49 | training |
| 747 | 1567 | 2.10 | 38.75 | 3999.82 | 16.12 | 185.78 | 44.71 | 193.47 | 6.33 | 9.84 | 2.71 | 10.85 | training |
| 758 | 1549 | 2.04 | 38.94 | 4104.70 | 16.13 | 169.02 | 49.06 | 260.70 | 5.54 | 9.48 | 2.44 | 10.83 | training |
| 670 | 1700 | 2.50 | 40.04 | 4445.47 | 16.67 | 189.86 | 54.77 | 289.21 | 4.44 | 9.29 | 2.52 | 12.01 | training |
| 670 | 1790 | 2.63 | 40.04 | 4541.39 | 17.07 | 197.87 | 93.29 | 307.35 | 4.62 | 9.54 | 2.77 | 12.51 | training |
| 703 | 1485 | 2.11 | 41.84 | 3672.31 | 17.08 | 182.94 | 57.01 | 294.66 | 4.99 | 9.53 | 2.48 | 12.10 | training |
| 731 | 1547 | 2.12 | 42.50 | 3851.63 | 16.58 | 213.15 | 77.22 | 184.99 | 4.79 | 7.53 | 2.06 | 8.33 | training |
| 989 | 1853 | 1.87 | 46.60 | 4810.88 | 25.12 | 166.77 | 50.70 | 357.20 | 9.43 | 14.86 | 5.30 | 18.78 | training |
| 989 | 1853 | 1.87 | 46.60 | 4812.14 | 25.41 | 148.95 | 72.94 | 351.70 | 8.25 | 12.70 | 3.80 | 15.36 | training |
| 840 | 2050 | 2.44 | 47.45 | 5216.78 | 20.21 | 238.57 | 66.75 | 344.81 | 6.71 | 12.26 | 3.28 | 14.35 | training |
| 891 | 2019 | 2.27 | 48.55 | 5073.16 | 19.72 | 268.46 | 96.50 | 346.81 | 7.58 | 12.29 | 3.41 | 15.83 | training |
| 929 | 2093 | 2.24 | 48.95 | 5400.64 | 24.65 | 189.57 | 54.04 | 431.88 | 10.38 | 15.88 | 4.19 | 18.51 | training |
| 929 | 2093 | 2.25 | 48.95 | 5398.67 | 21.92 | 233.49 | 63.31 | 311.92 | 9.16 | 13.51 | 4.03 | 17.30 | training |
| 924 | 2164 | 2.37 | 50.07 | 5754.02 | 21.74 | 258.16 | 97.09 | 346.12 | 6.11 | 11.01 | 3.04 | 14.58 | training |
| 930 | 1950 | 2.08 | 51.20 | 5308.82 | 17.80 | 212.92 | 55.34 | 295.56 | 4.43 | 9.32 | 2.42 | 12.21 | training |
| 1032 | 2304 | 2.23 | 51.43 | 5894.26 | 22.57 | 282.48 | 106.64 | 393.73 | 6.57 | 12.63 | 3.51 | 16.59 | training |
| 950 | 1920 | 2.04 | 51.55 | 5191.00 | 19.48 | 210.38 | 56.64 | 320.60 | 4.65 | 10.31 | 2.72 | 13.27 | training |
| 960 | 2280 | 2.35 | 52.95 | 5817.09 | 21.88 | 271.08 | 102.16 | 366.62 | 5.99 | 11.68 | 3.24 | 15.44 | training |
| 997 | 2378 | 2.37 | 53.20 | 6307.32 | 23.22 | 287.84 | 105.25 | 355.14 | 6.74 | 11.06 | 3.02 | 14.93 | training |
| 1025 | 2722 | 2.65 | 55.75 | 7061.14 | 20.58 | 416.04 | 190.34 | 524.16 | 7.03 | 15.21 | 5.28 | 21.06 | training |
| 747 | 2251 | 3.01 | 59.76 | 5850.24 | 16.66 | 272.87 | 60.58 | 231.28 | 6.75 | 10.53 | 2.93 | 11.38 | training |
| 778 | 2261 | 2.91 | 60.19 | 5871.22 | 16.73 | 273.88 | 60.80 | 232.17 | 6.78 | 10.57 | 2.94 | 13.23 | training |
| 786 | 2340 | 2.98 | 60.30 | 6078.95 | 17.31 | 283.55 | 62.95 | 240.35 | 7.02 | 10.94 | 3.05 | 12.76 | training |
| 740 | 2045 | 2.76 | 60.45 | 5579.52 | 16.34 | 216.47 | 60.66 | 387.22 | 5.84 | 12.37 | 3.17 | 15.82 | training |
| 810 | 2454 | 3.03 | 61.65 | 5890.56 | 21.59 | 303.02 | 106.78 | 397.62 | 7.22 | 14.10 | 3.46 | 18.99 | training |
| 696 | 1988 | 2.86 | 61.94 | 5372.88 | 17.33 | 211.46 | 79.07 | 421.46 | 6.66 | 13.61 | 3.74 | 17.12 | training |
| 740 | 2750 | 3.70 | 62.34 | 6714.99 | 22.06 | 301.28 | 87.36 | 463.69 | 7.10 | 14.92 | 4.05 | 19.26 | training |
| 733 | 1972 | 2.69 | 62.46 | 5321.45 | 17.19 | 213.13 | 62.44 | 352.29 | 6.33 | 11.38 | 3.07 | 14.47 | training |
| 743 | 1967 | 2.65 | 62.60 | 5309.59 | 17.15 | 212.65 | 62.29 | 351.45 | 5.92 | 11.35 | 3.07 | 14.44 | training |
| 760 | 2330 | 3.03 | 62.62 | 6126.33 | 19.98 | 259.67 | 77.87 | 373.86 | 5.82 | 11.82 | 3.19 | 15.47 | training |
| 790 | 2510 | 3.13 | 63.04 | 6398.79 | 20.94 | 277.50 | 130.05 | 414.48 | 6.30 | 12.73 | 3.68 | 16.83 | training |
| 790 | 2570 | 3.23 | 63.04 | 6412.30 | 20.97 | 281.71 | 178.84 | 434.22 | 6.46 | 12.96 | 3.99 | 17.32 | training |
| 780 | 2160 | 2.78 | 65.41 | 5501.98 | 15.10 | 264.12 | 69.46 | 339.49 | 5.24 | 10.69 | 3.10 | 14.17 | training |
| 830 | 2130 | 2.63 | 66.34 | 5560.25 | 14.84 | 242.17 | 66.00 | 329.84 | 5.16 | 10.40 | 2.84 | 13.69 | training |
| 830 | 2130 | 2.57 | 66.34 | 5535.19 | 14.88 | 237.33 | 81.62 | 332.16 | 5.19 | 10.32 | 2.82 | 13.65 | training |
| 870 | 2060 | 2.38 | 66.85 | 5548.97 | 14.21 | 224.13 | 62.10 | 313.79 | 4.93 | 9.90 | 2.64 | 13.00 | training |
| 780 | 2110 | 2.70 | 66.97 | 5416.16 | 14.62 | 243.76 | 65.92 | 327.14 | 5.13 | 10.29 | 2.82 | 13.59 | training |
| 780 | 2110 | 2.71 | 66.97 | 5385.16 | 14.68 | 233.27 | 110.52 | 337.07 | 5.16 | 10.24 | 2.96 | 13.65 | training |
| 940 | 2790 | 2.94 | 67.97 | 7197.99 | 25.38 | 385.56 | 131.97 | 543.57 | 9.44 | 17.42 | 4.87 | 22.28 | training |
| 830 | 2100 | 2.53 | 68.22 | 5554.02 | 14.61 | 231.87 | 76.96 | 326.71 | 5.10 | 10.19 | 2.78 | 13.45 | training |
| 950 | 2160 | 2.27 | 68.50 | 5915.52 | 17.95 | 239.15 | 71.02 | 460.68 | 6.21 | 15.43 | 4.23 | 19.16 | training |
| 1000 | 2630 | 2.63 | 68.61 | 7880.42 | 28.80 | 288.38 | 78.82 | 411.49 | 9.93 | 12.94 | 3.32 | 16.86 | training |
| 960 | 2110 | 2.17 | 68.90 | 5682.70 | 22.69 | 231.93 | 64.16 | 378.27 | 6.00 | 12.33 | 3.30 | 15.68 | training |
| 910 | 2620 | 2.86 | 69.32 | 7010.38 | 20.14 | 314.23 | 116.04 | 397.33 | 6.67 | 12.47 | 3.42 | 16.73 | training |
| 846 | 2664 | 3.14 | 69.44 | 6797.64 | 31.12 | 290.68 | 84.32 | 530.92 | 14.20 | 17.64 | 4.80 | 22.06 | training |
| 925 | 2819 | 3.05 | 71.14 | 7193.15 | 57.74 | 297.13 | 101.43 | 445.85 | 7.93 | 13.96 | 3.84 | 18.28 | training |
| 948 | 2833 | 2.99 | 71.40 | 7522.89 | 49.66 | 386.96 | 142.09 | 410.82 | 6.46 | 11.37 | 2.66 | 16.09 | training |
| 939 | 2581 | 2.75 | 71.47 | 6643.66 | 34.31 | 356.32 | 135.88 | 386.45 | 8.34 | 13.48 | 3.60 | 15.14 | training |
| 989 | 2770 | 2.76 | 72.65 | 7133.16 | 20.83 | 340.51 | 126.51 | 450.87 | 6.86 | 14.29 | 3.94 | 18.98 | training |
| 799 | 2083 | 2.61 | 73.09 | 5185.32 | 23.12 | 286.48 | 80.44 | 330.45 | 5.30 | 10.90 | 2.89 | 14.21 | training |
| 813 | 2550 | 3.14 | 73.30 | 6599.59 | 23.14 | 294.75 | 76.85 | 421.51 | 8.65 | 13.50 | 3.72 | 17.51 | training |
| 813 | 2550 | 3.13 | 73.30 | 6542.62 | 24.42 | 278.65 | 97.43 | 431.21 | 9.77 | 13.69 | 3.73 | 17.70 | training |
| 745 | 2283 | 3.07 | 73.49 | 6257.81 | 14.04 | 242.13 | 64.46 | 383.78 | 6.06 | 11.93 | 2.96 | 15.61 | training |
| 1000 | 2798 | 2.79 | 74.16 | 7468.20 | 20.96 | 339.50 | 128.39 | 454.99 | 6.89 | 14.51 | 4.02 | 19.20 | training |
| 813 | 2121 | 2.61 | 74.68 | 5282.37 | 21.74 | 270.75 | 82.79 | 424.43 | 8.82 | 15.42 | 4.45 | 18.25 | training |
| 850 | 2369 | 2.79 | 76.73 | 6703.40 | 16.45 | 256.79 | 69.57 | 423.93 | 6.58 | 13.32 | 3.35 | 17.28 | training |
| 959 | 2786 | 2.91 | 76.99 | 7419.78 | 20.07 | 338.35 | 122.89 | 393.04 | 7.12 | 12.05 | 3.26 | 16.50 | training |
| 990 | 2900 | 2.93 | 80.70 | 7566.70 | 18.51 | 358.92 | 77.79 | 286.41 | 7.68 | 12.81 | 2.83 | 13.32 | training |
| 697 | 2806 | 4.03 | 80.86 | 7354.88 | 17.23 | 345.46 | 71.71 | 241.46 | 7.30 | 11.32 | 4.00 | 10.81 | training |
| 748 | 2927 | 3.91 | 81.57 | 7669.61 | 17.98 | 360.26 | 74.78 | 251.80 | 7.62 | 11.80 | 4.17 | 12.15 | training |
| 764 | 2977 | 3.90 | 81.80 | 7798.15 | 18.28 | 366.31 | 76.03 | 256.03 | 7.75 | 12.00 | 4.24 | 13.25 | training |
| 1050 | 2850 | 2.71 | 81.95 | 7434.66 | 18.19 | 352.67 | 76.44 | 281.42 | 7.55 | 12.59 | 3.35 | 13.09 | training |
| 776 | 2979 | 3.84 | 81.96 | 7800.92 | 18.29 | 366.46 | 76.06 | 256.13 | 7.75 | 12.01 | 4.24 | 14.15 | training |
| 1050 | 2850 | 2.71 | 82.30 | 7433.08 | 18.18 | 352.61 | 76.42 | 281.38 | 7.55 | 12.59 | 3.92 | 13.09 | training |
| 850 | 2480 | 2.92 | 83.55 | 6466.71 | 15.63 | 306.77 | 66.49 | 244.81 | 6.81 | 11.45 | 2.92 | 11.63 | training |
| 900 | 2610 | 2.90 | 84.20 | 6807.86 | 16.45 | 322.94 | 69.99 | 257.70 | 7.17 | 11.27 | 3.07 | 12.25 | training |
| 840 | 3070 | 3.65 | 86.25 | 8012.56 | 19.64 | 362.58 | 90.25 | 405.69 | 6.65 | 12.26 | 3.33 | 16.81 | training |
| 727 | 2677 | 3.69 | 86.96 | 7388.41 | 21.57 | 289.23 | 84.74 | 478.17 | 9.12 | 15.44 | 4.17 | 19.65 | training |
| 850 | 2550 | 3.00 | 87.68 | 6601.87 | 15.65 | 309.20 | 68.47 | 261.02 | 6.09 | 10.37 | 2.57 | 11.07 | training |
| 880 | 2700 | 3.07 | 88.20 | 7000.66 | 20.35 | 307.93 | 83.34 | 411.52 | 6.51 | 12.89 | 3.45 | 17.06 | training |
| 910 | 2640 | 2.90 | 88.73 | 6819.30 | 21.56 | 320.11 | 70.89 | 270.23 | 9.74 | 14.43 | 3.72 | 14.89 | training |
| 767 | 2657 | 3.46 | 88.83 | 7324.38 | 21.41 | 275.15 | 68.73 | 357.64 | 6.78 | 10.99 | 2.82 | 14.68 | training |
| 920 | 2680 | 2.91 | 88.90 | 6930.53 | 19.17 | 324.96 | 71.96 | 274.33 | 8.28 | 12.77 | 3.24 | 13.24 | training |
| 778 | 2628 | 3.38 | 89.34 | 7245.90 | 21.17 | 272.21 | 67.99 | 353.78 | 6.44 | 10.87 | 2.79 | 14.52 | training |
| 619 | 2504 | 4.08 | 89.54 | 6713.14 | 11.17 | 329.56 | 103.72 | 349.48 | 5.42 | 10.30 | 2.63 | 14.14 | training |
| 890 | 2780 | 3.12 | 90.80 | 7307.02 | 20.39 | 304.32 | 69.34 | 267.96 | 9.58 | 13.00 | 3.46 | 14.60 | training |
| 920 | 2840 | 3.09 | 91.26 | 7475.73 | 20.84 | 311.34 | 70.93 | 274.06 | 9.79 | 13.28 | 3.54 | 14.92 | training |
| 920 | 3100 | 3.33 | 91.49 | 8058.88 | 16.02 | 371.43 | 91.15 | 404.21 | 6.62 | 12.17 | 3.38 | 16.77 | training |
| 656 | 2530 | 3.86 | 91.98 | 6991.12 | 11.52 | 322.49 | 107.32 | 350.57 | 6.00 | 9.78 | 2.30 | 13.83 | training |
| 780 | 3380 | 4.35 | 92.00 | 8264.35 | 23.68 | 370.42 | 104.11 | 534.51 | 8.33 | 16.98 | 4.55 | 22.17 | training |
| 780 | 3200 | 4.00 | 92.00 | 8185.24 | 23.05 | 347.77 | 239.79 | 487.51 | 7.39 | 13.89 | 4.38 | 19.14 | training |
| 980 | 2960 | 3.03 | 92.12 | 7863.85 | 15.99 | 330.83 | 84.79 | 390.48 | 6.43 | 11.85 | 3.07 | 16.12 | training |
| 980 | 2960 | 3.02 | 92.12 | 7848.82 | 16.02 | 326.73 | 114.04 | 391.47 | 6.40 | 11.59 | 3.10 | 15.93 | training |
| 950 | 2980 | 3.13 | 92.22 | 7831.64 | 15.72 | 345.36 | 86.49 | 390.50 | 6.41 | 11.80 | 3.17 | 16.17 | training |
| 950 | 2980 | 3.14 | 92.22 | 7772.90 | 15.83 | 329.11 | 135.82 | 394.71 | 6.41 | 11.47 | 3.17 | 15.91 | training |
| 1000 | 2940 | 2.94 | 92.35 | 7935.69 | 16.01 | 324.14 | 83.62 | 387.87 | 6.38 | 11.81 | 3.05 | 16.01 | training |
| 1000 | 2940 | 2.94 | 92.35 | 7925.67 | 16.03 | 324.46 | 91.35 | 388.75 | 6.40 | 11.74 | 3.03 | 15.98 | training |
| 1020 | 2900 | 2.86 | 92.89 | 7984.77 | 15.80 | 320.27 | 82.25 | 381.55 | 6.23 | 11.64 | 3.08 | 15.78 | training |
| 1003 | 3580 | 3.57 | 93.57 | 9283.93 | 21.68 | 440.32 | 158.26 | 509.17 | 8.16 | 15.64 | 4.22 | 21.42 | training |
| 717 | 2700 | 3.77 | 94.09 | 6997.33 | 14.59 | 317.99 | 72.51 | 530.95 | 15.19 | 18.39 | 5.07 | 22.26 | training |
| 1019 | 3662 | 3.60 | 95.35 | 9842.22 | 22.07 | 444.85 | 162.45 | 520.12 | 8.30 | 16.08 | 4.37 | 21.95 | training |
| 1046 | 3013 | 2.88 | 95.57 | 7833.18 | 35.79 | 445.25 | 178.84 | 429.67 | 10.53 | 12.32 | 2.88 | 16.01 | training |
| 1046 | 3013 | 2.88 | 95.57 | 7833.37 | 35.79 | 445.37 | 179.29 | 427.59 | 8.14 | 14.35 | 3.76 | 15.93 | training |
| 968 | 2728 | 2.82 | 96.90 | 7013.49 | 26.41 | 301.60 | 74.27 | 382.35 | 9.03 | 12.09 | 3.14 | 15.90 | training |
| 952 | 2870 | 3.01 | 97.43 | 7481.11 | 31.00 | 395.80 | 149.49 | 437.12 | 10.07 | 13.28 | 3.49 | 16.84 | training |
| 996 | 2776 | 2.79 | 97.45 | 7269.68 | 24.27 | 308.75 | 80.37 | 430.36 | 16.99 | 15.53 | 9.36 | 17.78 | training |
| 762 | 2932 | 3.83 | 97.55 | 7374.74 | 19.15 | 305.33 | 90.15 | 424.14 | 6.62 | 13.11 | 3.50 | 17.38 | training |
| 715 | 2590 | 3.62 | 97.55 | 6776.61 | 20.83 | 304.43 | 76.14 | 391.73 | 7.90 | 12.30 | 3.42 | 16.27 | training |
| 715 | 2590 | 3.57 | 97.55 | 6695.79 | 22.12 | 283.20 | 105.97 | 431.68 | 8.82 | 13.58 | 3.73 | 17.66 | training |
| 930 | 2540 | 2.70 | 97.60 | 6995.24 | 19.31 | 276.46 | 68.71 | 338.07 | 5.32 | 10.34 | 2.60 | 13.92 | training |
| 950 | 2540 | 2.70 | 97.90 | 6961.84 | 21.12 | 277.20 | 70.72 | 365.52 | 5.59 | 11.40 | 2.92 | 15.08 | training |
| 1017 | 3385 | 3.33 | 98.65 | 8916.53 | 19.56 | 381.32 | 101.69 | 566.92 | 8.30 | 18.15 | 4.77 | 23.46 | training |
| 747 | 2369 | 3.17 | 99.42 | 5952.81 | 25.84 | 316.02 | 87.14 | 355.29 | 5.17 | 11.11 | 3.01 | 15.23 | training |
| 932 | 3420 | 3.67 | 101.05 | 8992.69 | 16.59 | 415.15 | 86.34 | 290.44 | 7.16 | 11.96 | 3.08 | 13.00 | training |
| 738 | 2453 | 3.32 | 101.11 | 6208.82 | 20.72 | 345.38 | 93.74 | 369.35 | 7.98 | 14.51 | 3.72 | 17.19 | training |
| 998 | 3380 | 3.39 | 101.97 | 8842.05 | 22.46 | 410.29 | 85.33 | 287.05 | 10.79 | 15.88 | 4.06 | 16.90 | training |
| 1010 | 3210 | 3.18 | 102.14 | 8406.81 | 19.32 | 389.66 | 81.04 | 272.61 | 8.96 | 14.12 | 3.53 | 15.09 | training |
| 1010 | 3170 | 3.14 | 102.14 | 8273.55 | 20.28 | 374.39 | 93.19 | 418.91 | 6.86 | 12.66 | 3.44 | 17.36 | training |
| 769 | 2683 | 3.48 | 103.47 | 6748.44 | 36.30 | 341.77 | 126.45 | 424.51 | 7.60 | 12.49 | 3.18 | 16.97 | training |
| 960 | 3250 | 3.39 | 105.90 | 8570.10 | 16.29 | 396.61 | 82.54 | 277.58 | 6.84 | 11.72 | 3.89 | 12.74 | training |
| 970 | 3260 | 3.36 | 106.08 | 8597.82 | 16.34 | 397.89 | 82.81 | 278.47 | 6.86 | 11.76 | 3.42 | 12.78 | training |
| 776 | 2979 | 3.84 | 106.50 | 7633.25 | 22.02 | 269.85 | 76.92 | 614.72 | 14.78 | 22.61 | 5.96 | 26.35 | training |
| 1000 | 3180 | 3.18 | 106.60 | 8388.15 | 15.94 | 388.18 | 80.79 | 271.68 | 6.70 | 11.47 | 2.86 | 12.47 | training |
| 943 | 3667 | 3.89 | 107.00 | 9905.86 | 18.59 | 386.17 | 80.99 | 271.32 | 7.79 | 12.79 | 3.53 | 13.31 | training |
| 951 | 3595 | 3.78 | 107.11 | 9705.39 | 18.22 | 378.35 | 79.35 | 265.82 | 7.64 | 12.53 | 3.46 | 15.19 | training |
| 961 | 3613 | 3.76 | 107.25 | 9756.98 | 18.31 | 380.36 | 79.77 | 267.23 | 7.68 | 12.60 | 3.48 | 14.19 | training |
| 1040 | 3310 | 3.18 | 108.04 | 8759.42 | 22.10 | 319.81 | 90.05 | 582.78 | 23.07 | 24.05 | 10.75 | 27.84 | training |
| 833 | 3292 | 3.95 | 108.55 | 8164.52 | 16.64 | 577.81 | 168.80 | 552.56 | 7.77 | 16.49 | 4.93 | 22.75 | training |
| 880 | 2960 | 3.36 | 108.92 | 8003.11 | 15.02 | 317.81 | 65.42 | 221.21 | 6.38 | 10.85 | 2.67 | 11.27 | training |
| 900 | 3090 | 3.43 | 109.20 | 8352.03 | 15.68 | 331.67 | 68.27 | 230.85 | 7.58 | 11.33 | 2.79 | 11.76 | training |
| 900 | 2980 | 3.31 | 109.20 | 8059.66 | 15.12 | 320.06 | 65.88 | 222.78 | 5.53 | 10.93 | 2.69 | 11.35 | training |
| 949 | 3426 | 3.61 | 109.50 | 8401.87 | 22.20 | 603.57 | 182.59 | 646.21 | 8.89 | 19.88 | 5.99 | 26.70 | training |
| 1200 | 3170 | 2.61 | 110.20 | 8231.12 | 29.55 | 355.30 | 87.07 | 416.62 | 6.67 | 12.61 | 3.13 | 17.14 | training |
| 893 | 2980 | 3.33 | 113.05 | 7236.99 | 20.99 | 575.49 | 150.60 | 470.69 | 6.12 | 13.90 | 4.39 | 19.78 | training |
| 1040 | 3390 | 3.22 | 116.20 | 8977.86 | 24.02 | 383.21 | 103.02 | 581.62 | 9.81 | 18.70 | 4.94 | 24.08 | training |
| 860 | 2840 | 3.30 | 116.40 | 6905.46 | 16.95 | 548.81 | 143.69 | 449.82 | 5.85 | 13.29 | 4.20 | 18.91 | training |
| 770 | 2580 | 3.34 | 117.50 | 6920.91 | 19.64 | 288.76 | 73.15 | 509.06 | 15.34 | 18.07 | 4.78 | 21.27 | training |
| 860 | 2720 | 3.15 | 117.95 | 7330.07 | 20.39 | 296.25 | 65.24 | 240.51 | 12.42 | 8.02 | 5.70 | 14.12 | training |
| 1150 | 3190 | 2.74 | 118.13 | 8309.99 | 29.73 | 359.24 | 89.30 | 439.55 | 6.91 | 13.45 | 3.38 | 18.10 | training |
| 900 | 2760 | 3.05 | 118.40 | 7511.74 | 19.59 | 301.48 | 70.88 | 310.02 | 9.38 | 9.85 | 3.52 | 14.36 | training |
| 910 | 2840 | 3.10 | 118.55 | 7691.53 | 20.72 | 309.68 | 70.48 | 284.62 | 11.31 | 9.38 | 4.93 | 14.74 | training |
| 223 | 269 | 1.21 | 5.57 | 719.11 | 4.44 | 15.19 | 4.39 | 57.68 | 1.21 | 2.25 | 0.69 | 2.66 | testing |
| 230 | 319 | 1.39 | 7.01 | 837.00 | 4.64 | 18.13 | 5.61 | 74.04 | 1.71 | 2.89 | 0.88 | 3.40 | testing |
| 267 | 349 | 1.31 | 7.29 | 932.11 | 5.69 | 25.02 | 6.74 | 69.09 | 1.56 | 2.64 | 0.77 | 3.14 | testing |
| 258 | 341 | 1.33 | 8.05 | 861.93 | 4.79 | 32.95 | 9.28 | 60.04 | 2.38 | 2.31 | 1.11 | 2.87 | testing |
| 200 | 318 | 1.59 | 8.30 | 768.01 | 4.45 | 30.68 | 15.84 | 67.31 | 1.84 | 2.30 | 0.67 | 2.81 | testing |
| 269 | 370 | 1.38 | 8.34 | 992.60 | 4.74 | 20.55 | 6.31 | 84.11 | 3.40 | 4.06 | 1.00 | 3.87 | testing |
| 210 | 313 | 1.49 | 8.35 | 776.02 | 4.33 | 30.65 | 8.94 | 66.37 | 1.77 | 2.33 | 0.64 | 2.82 | testing |
| 273 | 373 | 1.37 | 8.38 | 997.50 | 5.22 | 28.58 | 8.99 | 75.69 | 2.07 | 2.72 | 0.78 | 3.29 | testing |
| 275 | 465 | 1.69 | 8.55 | 1165.45 | 6.47 | 44.19 | 33.05 | 98.26 | 2.74 | 3.26 | 1.00 | 4.03 | testing |
| 312 | 421 | 1.35 | 8.80 | 1127.47 | 5.10 | 32.25 | 10.19 | 92.16 | 4.17 | 3.57 | 1.94 | 4.17 | testing |
| 320 | 449 | 1.41 | 8.89 | 1147.06 | 5.64 | 34.44 | 10.87 | 98.31 | 4.45 | 3.80 | 2.07 | 4.45 | testing |
| 203 | 372 | 1.83 | 8.92 | 974.29 | 4.97 | 33.91 | 9.84 | 75.34 | 1.58 | 2.74 | 0.79 | 3.34 | testing |
| 165 | 305 | 1.85 | 9.02 | 769.11 | 4.14 | 40.54 | 11.35 | 59.23 | 1.12 | 1.99 | 0.56 | 2.52 | testing |
| 338 | 425 | 1.25 | 9.02 | 1132.30 | 5.70 | 38.79 | 11.32 | 93.23 | 1.75 | 3.34 | 0.91 | 4.00 | testing |
| 365 | 460 | 1.27 | 9.30 | 1221.34 | 7.36 | 41.26 | 11.52 | 91.98 | 3.46 | 3.46 | 1.39 | 3.95 | testing |
| 387 | 477 | 1.23 | 9.38 | 1215.16 | 7.23 | 36.75 | 11.59 | 104.56 | 4.73 | 4.05 | 2.20 | 4.73 | testing |
| 375 | 469 | 1.25 | 9.43 | 1194.13 | 7.72 | 36.11 | 11.39 | 102.79 | 4.65 | 3.98 | 2.16 | 4.65 | testing |
| 170 | 362 | 2.16 | 10.45 | 878.49 | 4.89 | 49.59 | 13.85 | 72.06 | 1.76 | 2.47 | 0.72 | 3.11 | testing |
| 395 | 464 | 1.45 | 11.02 | 1143.87 | 6.18 | 52.24 | 13.86 | 91.08 | 1.24 | 3.00 | 0.84 | 3.80 | testing |
| 386 | 551 | 1.42 | 11.22 | 1459.74 | 10.03 | 54.32 | 11.33 | 95.26 | 4.34 | 3.41 | 2.20 | 4.63 | testing |
| 443 | 545 | 1.23 | 11.44 | 1430.79 | 9.41 | 54.71 | 14.36 | 112.80 | 4.33 | 4.19 | 1.80 | 4.94 | testing |
| 392 | 613 | 1.56 | 12.17 | 1635.60 | 8.47 | 60.44 | 16.29 | 118.32 | 3.76 | 5.29 | 1.07 | 5.03 | testing |
| 499 | 757 | 1.49 | 12.50 | 1994.44 | 9.82 | 84.73 | 21.46 | 149.36 | 3.74 | 5.30 | 1.40 | 6.24 | testing |
| 500 | 741 | 1.47 | 12.52 | 1953.25 | 9.61 | 82.93 | 21.01 | 146.21 | 3.37 | 5.19 | 1.37 | 6.11 | testing |
| 369 | 609 | 1.65 | 13.13 | 1579.11 | 7.57 | 54.81 | 16.66 | 117.40 | 3.10 | 4.88 | 1.74 | 6.17 | testing |
| 395 | 652 | 1.65 | 13.62 | 1669.80 | 8.61 | 75.52 | 27.86 | 116.99 | 4.25 | 5.84 | 1.80 | 6.75 | testing |
| 405 | 630 | 1.56 | 13.75 | 1674.86 | 6.82 | 61.93 | 18.41 | 119.24 | 3.97 | 4.61 | 1.27 | 5.18 | testing |
| 430 | 631 | 1.47 | 14.15 | 1668.18 | 7.85 | 62.45 | 17.92 | 113.17 | 2.39 | 4.12 | 1.13 | 4.92 | testing |
| 431 | 601 | 1.39 | 14.15 | 1591.31 | 8.31 | 65.44 | 25.80 | 77.23 | 2.48 | 2.38 | 0.64 | 3.14 | testing |
| 446 | 690 | 1.55 | 14.33 | 1825.58 | 8.98 | 69.31 | 19.56 | 122.74 | 4.25 | 6.00 | 1.85 | 6.75 | testing |
| 397 | 629 | 1.58 | 14.35 | 1624.81 | 8.26 | 58.99 | 16.58 | 110.95 | 4.55 | 3.97 | 2.49 | 4.51 | testing |
| 397 | 629 | 1.58 | 14.35 | 1624.81 | 8.26 | 58.99 | 16.58 | 110.95 | 4.55 | 3.97 | 2.49 | 4.51 | testing |
| 448 | 619 | 1.38 | 14.35 | 1601.96 | 9.74 | 60.84 | 17.13 | 106.89 | 4.40 | 4.26 | 1.62 | 4.84 | testing |
| 573 | 986 | 1.72 | 14.35 | 2587.69 | 11.96 | 95.73 | 28.31 | 177.77 | 4.22 | 6.14 | 1.69 | 7.56 | testing |
| 458 | 636 | 1.39 | 14.50 | 1616.64 | 11.10 | 62.20 | 17.25 | 106.49 | 5.63 | 4.50 | 2.33 | 5.47 | testing |
| 412 | 640 | 1.55 | 14.56 | 1697.83 | 7.72 | 60.19 | 16.90 | 113.00 | 3.16 | 5.00 | 1.51 | 5.37 | testing |
| 428 | 800 | 1.86 | 14.85 | 1915.41 | 10.96 | 141.44 | 74.08 | 183.87 | 5.06 | 5.45 | 1.50 | 6.93 | testing |
| 442 | 772 | 1.75 | 15.06 | 1948.99 | 8.60 | 76.88 | 22.38 | 141.38 | 2.29 | 5.31 | 1.33 | 6.01 | testing |
| 442 | 772 | 1.75 | 15.06 | 1948.99 | 8.60 | 76.88 | 22.38 | 141.38 | 2.29 | 5.31 | 1.33 | 6.01 | testing |
| 583 | 932 | 1.59 | 15.08 | 2441.84 | 11.34 | 127.30 | 46.75 | 135.15 | 2.13 | 3.74 | 0.87 | 5.29 | testing |
| 446 | 813 | 1.82 | 15.25 | 1829.35 | 12.97 | 127.08 | 62.15 | 184.90 | 4.67 | 5.73 | 1.58 | 7.19 | testing |
| 569 | 861 | 1.51 | 15.65 | 2083.78 | 11.71 | 95.67 | 46.37 | 175.96 | 4.75 | 5.66 | 1.63 | 7.18 | testing |
| 532 | 1059 | 1.97 | 15.83 | 2706.63 | 11.67 | 142.39 | 46.79 | 206.61 | 3.45 | 6.69 | 1.87 | 8.53 | testing |
| 585 | 878 | 1.50 | 15.85 | 2209.87 | 11.72 | 83.44 | 62.41 | 185.53 | 5.17 | 6.15 | 1.88 | 7.62 | testing |
| 578 | 880 | 1.52 | 15.90 | 2163.18 | 11.89 | 98.20 | 37.99 | 179.86 | 4.83 | 5.88 | 1.65 | 7.41 | testing |
| 545 | 1042 | 1.91 | 16.47 | 2655.97 | 11.89 | 137.66 | 49.11 | 217.89 | 4.78 | 7.18 | 2.04 | 8.98 | testing |
| 558 | 1032 | 1.85 | 16.54 | 2633.98 | 11.52 | 131.07 | 39.15 | 201.79 | 3.20 | 6.67 | 1.86 | 8.45 | testing |
| 454 | 798 | 1.76 | 16.88 | 1984.43 | 9.93 | 85.28 | 32.80 | 156.98 | 3.15 | 6.18 | 1.72 | 7.08 | testing |
| 477 | 970 | 2.01 | 17.24 | 2409.36 | 11.06 | 129.96 | 38.00 | 214.88 | 3.84 | 7.46 | 2.19 | 9.24 | testing |
| 491 | 925 | 1.88 | 17.39 | 2382.54 | 10.52 | 125.61 | 34.90 | 179.75 | 3.40 | 6.03 | 1.70 | 7.64 | testing |
| 565 | 903 | 1.60 | 17.75 | 2214.63 | 12.21 | 100.77 | 38.98 | 184.52 | 4.95 | 6.03 | 1.69 | 7.60 | testing |
| 568 | 941 | 1.66 | 17.85 | 2337.83 | 12.63 | 105.70 | 30.63 | 192.55 | 5.08 | 6.39 | 1.74 | 8.00 | testing |
| 568 | 916 | 1.61 | 17.85 | 2216.90 | 12.45 | 101.57 | 49.29 | 187.13 | 5.05 | 6.02 | 1.74 | 7.63 | testing |
| 578 | 945 | 1.64 | 17.95 | 2378.46 | 12.61 | 85.53 | 25.80 | 218.14 | 7.97 | 8.22 | 3.20 | 9.35 | testing |
| 611 | 864 | 1.41 | 18.27 | 2355.17 | 11.56 | 66.81 | 17.87 | 164.98 | 3.83 | 5.61 | 1.62 | 6.76 | testing |
| 580 | 860 | 1.48 | 21.21 | 2151.21 | 9.39 | 95.13 | 36.38 | 172.37 | 11.30 | 5.64 | 1.57 | 7.12 | testing |
| 760 | 1190 | 1.57 | 24.19 | 3047.97 | 15.24 | 114.67 | 35.99 | 233.87 | 4.70 | 8.10 | 2.24 | 9.73 | testing |
| 738 | 1507 | 2.03 | 30.65 | 3825.10 | 12.97 | 168.14 | 46.58 | 275.28 | 3.90 | 8.98 | 2.40 | 11.41 | testing |
| 739 | 1632 | 2.19 | 34.05 | 4142.33 | 14.05 | 315.58 | 82.67 | 259.20 | 3.37 | 7.66 | 2.42 | 10.90 | testing |
| 459 | 1366 | 2.94 | 35.77 | 3509.32 | 11.29 | 166.46 | 63.03 | 234.82 | 3.63 | 7.55 | 2.10 | 9.89 | testing |
| 561 | 1450 | 2.58 | 36.84 | 3861.50 | 11.94 | 173.16 | 66.38 | 248.87 | 3.82 | 8.03 | 2.24 | 10.49 | testing |
| 810 | 2454 | 3.03 | 37.58 | 5890.56 | 21.59 | 303.02 | 106.78 | 397.62 | 7.22 | 14.10 | 3.70 | 18.99 | testing |
| 684 | 1534 | 2.24 | 37.61 | 3931.65 | 13.75 | 182.67 | 44.09 | 191.57 | 5.46 | 8.79 | 2.37 | 9.64 | testing |
| 675 | 1640 | 2.43 | 37.78 | 4349.98 | 17.80 | 160.99 | 44.08 | 256.36 | 7.76 | 8.29 | 2.21 | 10.61 | testing |
| 760 | 1450 | 1.89 | 37.91 | 3687.26 | 14.18 | 171.94 | 48.00 | 292.43 | 4.00 | 9.67 | 2.73 | 12.19 | testing |
| 706 | 1553 | 2.20 | 38.01 | 3979.40 | 13.92 | 184.84 | 44.62 | 193.88 | 5.52 | 8.89 | 2.78 | 9.76 | testing |
| 770 | 1470 | 1.91 | 38.01 | 3623.48 | 14.23 | 163.53 | 101.10 | 297.62 | 4.27 | 9.35 | 2.86 | 12.04 | testing |
| 800 | 1550 | 1.94 | 38.75 | 3746.05 | 15.27 | 134.58 | 34.98 | 244.87 | 13.04 | 10.32 | 7.54 | 13.50 | testing |
| 758 | 1549 | 2.04 | 38.94 | 4104.60 | 17.19 | 167.22 | 95.57 | 231.76 | 3.56 | 6.78 | 2.03 | 9.24 | testing |
| 735 | 1565 | 2.13 | 39.53 | 4339.05 | 18.83 | 172.47 | 49.97 | 314.18 | 4.30 | 10.43 | 2.84 | 13.05 | testing |
| 680 | 1920 | 2.78 | 40.18 | 4763.25 | 17.89 | 206.42 | 64.16 | 253.85 | 4.13 | 7.66 | 2.02 | 10.41 | testing |
| 823 | 1857 | 2.27 | 45.65 | 4329.83 | 16.72 | 203.81 | 49.77 | 236.39 | 4.04 | 7.13 | 1.77 | 9.72 | testing |
| 957 | 1963 | 2.05 | 45.80 | 5113.91 | 28.69 | 252.01 | 89.91 | 309.24 | 7.59 | 11.75 | 3.22 | 12.84 | testing |
| 789 | 1910 | 2.42 | 46.33 | 4927.67 | 19.07 | 217.67 | 57.53 | 321.01 | 7.30 | 10.32 | 2.82 | 13.33 | testing |
| 729 | 1910 | 2.63 | 46.33 | 4895.62 | 20.39 | 365.63 | 186.07 | 459.46 | 9.78 | 13.20 | 3.60 | 17.09 | testing |
| 840 | 1980 | 2.36 | 47.52 | 5094.12 | 19.41 | 221.84 | 55.06 | 243.74 | 7.65 | 11.83 | 3.27 | 13.42 | testing |
| 850 | 2020 | 2.38 | 47.94 | 5173.31 | 19.85 | 230.20 | 61.15 | 296.47 | 7.14 | 12.06 | 3.30 | 13.50 | testing |
| 910 | 2086 | 2.29 | 48.80 | 5099.51 | 18.90 | 321.91 | 136.28 | 405.69 | 7.15 | 11.93 | 3.24 | 15.67 | testing |
| 870 | 1950 | 2.22 | 50.25 | 5345.44 | 15.82 | 218.91 | 54.84 | 274.00 | 4.27 | 8.43 | 2.13 | 11.29 | testing |
| 970 | 1910 | 1.96 | 51.80 | 5128.10 | 21.31 | 210.01 | 58.50 | 348.43 | 4.92 | 11.39 | 3.05 | 14.45 | testing |
| 970 | 1910 | 1.96 | 51.85 | 5092.15 | 23.26 | 210.74 | 60.65 | 377.86 | 5.20 | 12.52 | 3.40 | 15.69 | testing |
| 1023 | 2490 | 2.45 | 51.91 | 6387.50 | 24.87 | 303.24 | 112.61 | 398.74 | 7.08 | 12.62 | 3.48 | 16.78 | testing |
| 776 | 2122 | 2.74 | 54.07 | 5491.00 | 18.70 | 195.90 | 58.56 | 386.95 | 12.66 | 13.80 | 3.64 | 16.10 | testing |
| 737 | 2264 | 3.07 | 59.62 | 5886.53 | 16.75 | 274.55 | 60.95 | 232.69 | 6.79 | 10.59 | 2.95 | 10.54 | testing |
| 820 | 1910 | 2.33 | 61.85 | 5379.61 | 17.31 | 213.11 | 59.04 | 348.90 | 4.94 | 11.38 | 3.04 | 14.47 | testing |
| 717 | 1976 | 2.75 | 62.24 | 5335.52 | 17.23 | 213.83 | 62.62 | 353.20 | 4.96 | 11.40 | 3.08 | 14.51 | testing |
| 780 | 2160 | 2.77 | 65.41 | 5411.56 | 15.27 | 239.55 | 85.29 | 415.54 | 6.14 | 13.55 | 3.81 | 17.22 | testing |
| 870 | 2060 | 2.37 | 66.85 | 5548.84 | 14.21 | 226.06 | 67.55 | 317.43 | 4.98 | 9.97 | 2.68 | 13.12 | testing |
| 830 | 2100 | 2.50 | 68.22 | 5563.81 | 14.59 | 233.16 | 64.19 | 323.00 | 5.07 | 10.19 | 2.74 | 13.39 | testing |
| 990 | 2740 | 2.78 | 68.39 | 7616.00 | 27.28 | 396.94 | 133.56 | 419.81 | 8.95 | 12.60 | 3.20 | 17.00 | testing |
| 970 | 2110 | 2.17 | 68.85 | 5746.65 | 19.26 | 230.64 | 60.36 | 326.27 | 4.97 | 10.33 | 2.69 | 13.49 | testing |
| 980 | 2190 | 2.22 | 69.00 | 5931.91 | 21.76 | 240.06 | 64.64 | 365.84 | 5.64 | 11.77 | 3.11 | 15.15 | testing |
| 845 | 2674 | 3.16 | 71.91 | 6869.34 | 19.35 | 317.47 | 117.29 | 398.45 | 7.52 | 12.48 | 3.42 | 16.77 | testing |
| 948 | 3032 | 3.18 | 72.60 | 7830.88 | 23.39 | 371.88 | 136.04 | 460.36 | 7.79 | 14.38 | 3.93 | 19.38 | testing |
| 1037 | 2648 | 2.55 | 73.77 | 6888.34 | 31.96 | 336.50 | 115.18 | 356.76 | 10.86 | 15.68 | 4.42 | 17.41 | testing |
| 1037 | 2648 | 2.55 | 73.77 | 6889.31 | 32.19 | 339.29 | 118.72 | 428.96 | 10.13 | 13.94 | 4.17 | 17.00 | testing |
| 1060 | 2980 | 2.81 | 82.60 | 7770.48 | 19.01 | 368.62 | 79.89 | 294.16 | 7.89 | 13.16 | 4.70 | 13.68 | testing |
| 850 | 2560 | 3.01 | 83.55 | 6681.69 | 16.14 | 316.93 | 68.69 | 252.89 | 7.04 | 9.52 | 3.01 | 12.02 | testing |
| 910 | 2680 | 2.95 | 84.33 | 6991.19 | 16.89 | 331.63 | 71.88 | 264.64 | 7.36 | 11.30 | 3.15 | 12.58 | testing |
| 800 | 2920 | 3.65 | 85.55 | 7542.35 | 17.53 | 342.95 | 71.60 | 240.41 | 7.48 | 10.56 | 3.07 | 14.55 | testing |
| 754 | 2663 | 3.53 | 88.11 | 7343.87 | 21.46 | 276.08 | 68.95 | 358.66 | 5.47 | 11.02 | 2.83 | 14.72 | testing |
| 890 | 2630 | 2.96 | 88.38 | 6785.70 | 23.94 | 318.90 | 70.62 | 269.21 | 11.28 | 16.22 | 4.23 | 16.68 | testing |
| 924 | 3522 | 3.76 | 90.49 | 9109.60 | 20.66 | 417.41 | 150.32 | 495.98 | 8.92 | 15.29 | 4.14 | 20.82 | testing |
| 920 | 3100 | 3.37 | 91.49 | 7955.03 | 16.22 | 337.28 | 93.46 | 472.21 | 7.42 | 14.90 | 3.97 | 19.57 | testing |
| 800 | 2970 | 3.70 | 92.28 | 7790.99 | 21.87 | 328.39 | 114.57 | 422.09 | 6.76 | 12.78 | 3.46 | 17.25 | testing |
| 1020 | 2900 | 2.84 | 92.89 | 7954.27 | 15.86 | 317.01 | 87.17 | 383.19 | 6.29 | 11.62 | 3.00 | 15.78 | testing |
| 870 | 2580 | 2.94 | 96.80 | 7171.11 | 15.95 | 284.36 | 80.78 | 494.55 | 8.42 | 16.30 | 4.40 | 20.53 | testing |
| 870 | 2520 | 2.86 | 97.00 | 6971.90 | 17.37 | 273.59 | 66.18 | 308.20 | 5.01 | 9.22 | 2.26 | 12.66 | testing |
| 952 | 2870 | 3.01 | 97.43 | 7480.96 | 30.93 | 395.20 | 145.65 | 372.43 | 10.36 | 15.10 | 4.17 | 17.27 | testing |
| 1005 | 3512 | 3.45 | 97.62 | 9414.78 | 20.48 | 430.53 | 152.63 | 461.50 | 7.98 | 13.89 | 3.70 | 19.40 | testing |
| 775 | 2981 | 3.85 | 97.74 | 7950.72 | 19.53 | 379.73 | 140.50 | 471.67 | 8.44 | 13.88 | 3.53 | 18.85 | testing |
| 980 | 2540 | 2.56 | 98.40 | 6929.85 | 22.92 | 277.88 | 72.72 | 392.92 | 5.98 | 12.44 | 3.24 | 16.24 | testing |
| 984 | 3370 | 3.42 | 101.78 | 8847.95 | 18.18 | 409.08 | 85.07 | 286.20 | 8.06 | 13.13 | 3.37 | 14.16 | testing |
| 994 | 3270 | 3.29 | 101.92 | 8542.50 | 23.51 | 396.94 | 82.55 | 277.71 | 11.75 | 17.00 | 4.58 | 17.66 | testing |
| 840 | 3050 | 3.63 | 108.36 | 8250.67 | 15.48 | 327.65 | 67.44 | 228.06 | 5.05 | 11.19 | 2.75 | 11.62 | testing |
| 1014 | 3575 | 3.53 | 110.10 | 8668.17 | 28.25 | 273.37 | 74.59 | 683.71 | 15.82 | 23.26 | 6.67 | 27.96 | testing |
| 942 | 3030 | 3.22 | 114.55 | 7349.39 | 24.59 | 332.36 | 89.14 | 501.46 | 7.61 | 16.10 | 4.25 | 20.76 | testing |
| 910 | 3150 | 3.50 | 116.10 | 8359.15 | 20.01 | 357.14 | 95.92 | 540.84 | 9.13 | 17.38 | 4.59 | 22.39 | testing |
| 1010 | 3320 | 3.23 | 116.13 | 9069.85 | 22.17 | 352.90 | 247.17 | 672.14 | 18.55 | 20.99 | 6.46 | 26.92 | testing |

^1^In total, 406 observations were obtained from the published articles. The nutrient intake was calculated by multiplying ADFI by nutrient concentrations of the corresponding treatment diet.

**Table S3.** Ingredients and nutrient compositions of the experimental diets in the animal trial (as-fed basis)

| Items, % | 100% NE req × 100% Lys req | 105% NE req  × 100% Lys req | 100% NE req × 130% Lys req | 105% NE req  × 130% Lys req |
| --- | --- | --- | --- | --- |
| Ingredients |  |  |  |  |
| Corn | 66.23 | 74.15 | 68.24 | 72.57 |
| Wheat middling | 10 | - | 7.2 | - |
| Soybean meal | 7.4 | 9.1 | 7.9 | 9.4 |
| Cottonseed meal | 4.1 | 4.1 | 4.1 | 4.1 |
| Cottonseed protein | 4 | 4 | 4 | 4 |
| Corn DDGS | 5 | 5 | 5 | 5 |
| L-lysine HCl | 0.36 | 0.34 | 0.57 | 0.56 |
| DL-methionine | 0.06 | 0.06 | 0.06 | 0.06 |
| L-threonine | 0.05 | 0.04 | 0.09 | 0.08 |
| L-tryptophan | - | - | 0.02 | 0.02 |
| Calcium phosphate | 0.72 | 0.79 | 0.74 | 0.8 |
| Limestone | 1 | 0.94 | 1 | 0.93 |
| Cottonseed oil | - | 0.4 | - | 1.4 |
| NaCl | 0.4 | 0.4 | 0.4 | 0.4 |
| Vitamin and mineral premix^1^ | 0.6 | 0.6 | 0.6 | 0.6 |
| Choline chloride | 0.08 | 0.08 | 0.08 | 0.08 |
| Total | 100 | 100 | 100 | 100 |
| Calculated compositions^2^, % |  |  |  |  |
| EE | 3.10 | 3.13 | 3.10 | 3.08 |
| Starch | 45.15 | 45.88 | 45.23 | 44.91 |
| NDF | 14.43 | 13.36 | 14.11 | 13.22 |
| ADF | 4.11 | 3.99 | 4.07 | 3.97 |
| CP | 15.19 | 15.11 | 15.16 | 15.11 |
| SID lysine | 0.87 | 0.86 | 1.09 | 1.09 |
| SID methionine | 0.29 | 0.29 | 0.29 | 0.29 |
| SID threonine | 0.48 | 0.48 | 0.51 | 0.51 |
| SID tryptophan | 0.13 | 0.13 | 0.14 | 0.14 |
| SID valine | 0.60 | 0.59 | 0.60 | 0.59 |
| NE, kcal/kg | 2496 | 2580 | 2496 | 2580 |

^1^Premix provided the following quantities per kilogram of diets: vitamin A as retinyl acetate, 8250 IU; vitamin D_3_ as cholecalciferol, 825 IU; vitamin E as DL-alpha-tocopheryl acetate, 40 IU; vitamin K_3_ as menadione nicotinamide bisulfite, 4 mg; vitamin B_12_, 25 μg; riboflavin, 5 mg; pantothenic acid as DL-calcium pantothenate, 15 mg; niacin, 35 mg; choline chloride, 600 mg; folacin, 2 mg; thiamin as thiamine mononitrate, 1 mg; pyridoxine as pyridoxine hydrochloride, 2 mg; biotin, 4 mg; Mn as MnO, 25 mg; Fe as FeSO_4_•H_2_O, 80 mg; Zn as ZnSO_4_, 100 mg; Cu as CuSO_4_•5H_2_O, 50 mg; I as KI, 0.5 mg; Se as Na_2_SeO_3_, 0.15 mg.

^2^ADF = acid detergent fiber, CP = crude protein, EE = ether extract, NDF = neutral detergent fiber, NE = net energy, SID = standardized ileal digestible. All the dietary nutrient concentrations were re-calculated based on the diet formulation and the nutrient compositions of ingredients published in *Nutrient Requirements of Swine in China* (National Standards of the People’s Republic of China, GB/T 39235-2020, released 2020-11-19).

**Table S4.** The validation sample obtained by the animal trial^1^

| Phase | Treatment | ADG, g/d | ADFI, g/d | F/G | BW, kg | NE intake, kcal/d | Lys intake, g/d |
| --- | --- | --- | --- | --- | --- | --- | --- |
| d1-14 | 1 | 790 | 1829 | 2.31 | 41.8 | 4565 | 15.98 |
| d1-14 | 1 | 888 | 2010 | 2.26 | 40.3 | 5018 | 17.57 |
| d1-14 | 1 | 742 | 1839 | 2.48 | 39.2 | 4590 | 16.07 |
| d1-14 | 1 | 785 | 1833 | 2.34 | 40.6 | 4576 | 16.02 |
| d1-14 | 2 | 840 | 1886 | 2.24 | 39.3 | 4794 | 16.29 |
| d1-14 | 2 | 771 | 1888 | 2.45 | 39.8 | 4800 | 16.31 |
| d1-14 | 2 | 815 | 1816 | 2.23 | 40.4 | 4616 | 15.68 |
| d1-14 | 2 | 739 | 1855 | 2.51 | 40.3 | 4716 | 16.02 |
| d1-14 | 3 | 824 | 1879 | 2.28 | 41.6 | 4689 | 20.42 |
| d1-14 | 3 | 826 | 1835 | 2.22 | 40.0 | 4578 | 19.94 |
| d1-14 | 3 | 708 | 1688 | 2.38 | 39.1 | 4211 | 18.34 |
| d1-14 | 3 | 824 | 1771 | 2.15 | 40.6 | 4421 | 19.26 |
| d1-14 | 4 | 883 | 1865 | 2.11 | 42.9 | 4812 | 20.31 |
| d1-14 | 4 | 811 | 1858 | 2.29 | 42.5 | 4794 | 20.24 |
| d1-14 | 4 | 792 | 1829 | 2.31 | 42.1 | 4718 | 19.92 |
| d1-14 | 4 | 937 | 1970 | 2.10 | 44.8 | 5082 | 21.45 |
| d14-28 | 1 | 819 | 1974 | 2.41 | 53.1 | 4928 | 17.26 |
| d14-28 | 1 | 846 | 2086 | 2.46 | 52.4 | 5208 | 18.23 |
| d14-28 | 1 | 1024 | 2291 | 2.24 | 51.6 | 5719 | 20.02 |
| d14-28 | 1 | 954 | 2197 | 2.30 | 52.8 | 5485 | 19.21 |
| d14-28 | 2 | 1054 | 2340 | 2.22 | 52.5 | 5948 | 20.21 |
| d14-28 | 2 | 886 | 2013 | 2.27 | 51.4 | 5118 | 17.39 |
| d14-28 | 2 | 890 | 2062 | 2.32 | 52.3 | 5241 | 17.80 |
| d14-28 | 2 | 1014 | 2177 | 2.15 | 52.5 | 5534 | 18.80 |
| d14-28 | 3 | 932 | 2182 | 2.34 | 53.9 | 5445 | 23.72 |
| d14-28 | 3 | 912 | 2034 | 2.23 | 52.2 | 5076 | 22.11 |
| d14-28 | 3 | 908 | 2061 | 2.27 | 50.4 | 5144 | 22.41 |
| d14-28 | 3 | 1039 | 2270 | 2.18 | 53.6 | 5665 | 24.67 |
| d14-28 | 4 | 1102 | 2427 | 2.20 | 56.8 | 6262 | 26.43 |
| d14-28 | 4 | 1006 | 2178 | 2.16 | 55.2 | 5618 | 23.71 |
| d14-28 | 4 | 1030 | 2159 | 2.10 | 54.8 | 5569 | 23.51 |
| d14-28 | 4 | 979 | 2368 | 2.42 | 58.3 | 6108 | 25.78 |
| d28-42 | 1 | 857 | 2211 | 2.58 | 64.8 | 5520 | 19.33 |
| d28-42 | 1 | 800 | 2310 | 2.89 | 64.5 | 5767 | 20.19 |
| d28-42 | 1 | 867 | 2545 | 2.94 | 64.8 | 6352 | 22.24 |
| d28-42 | 1 | 797 | 2226 | 2.79 | 64.8 | 5557 | 19.46 |
| d28-42 | 2 | 1014 | 2526 | 2.49 | 67.0 | 6422 | 21.82 |
| d28-42 | 2 | 788 | 2040 | 2.59 | 63.2 | 5186 | 17.62 |
| d28-42 | 2 | 836 | 2072 | 2.48 | 64.4 | 5268 | 17.90 |
| d28-42 | 2 | 813 | 2492 | 3.06 | 65.3 | 6335 | 21.52 |
| d28-42 | 3 | 829 | 2479 | 2.99 | 66.3 | 6185 | 26.94 |
| d28-42 | 3 | 733 | 2209 | 3.01 | 63.7 | 5513 | 24.02 |
| d28-42 | 3 | 768 | 2053 | 2.67 | 62.1 | 5124 | 22.32 |
| d28-42 | 3 | 893 | 2437 | 2.73 | 67.1 | 6082 | 26.49 |
| d28-42 | 4 | 967 | 2580 | 2.67 | 71.3 | 6657 | 28.10 |
| d28-42 | 4 | 813 | 2167 | 2.67 | 67.4 | 5591 | 23.60 |
| d28-42 | 4 | 727 | 2007 | 2.76 | 67.1 | 5177 | 21.85 |
| d28-42 | 4 | 835 | 2157 | 2.58 | 71.0 | 5563 | 23.48 |
| d42-56 | 1 | 1067 | 2762 | 2.59 | 78.3 | 6894 | 24.14 |
| d42-56 | 1 | 1177 | 3170 | 2.69 | 78.4 | 7914 | 27.71 |
| d42-56 | 1 | 1119 | 2971 | 2.66 | 78.7 | 7417 | 25.97 |
| d42-56 | 1 | 1021 | 2741 | 2.68 | 76.6 | 6841 | 23.95 |
| d42-56 | 2 | 1075 | 3092 | 2.88 | 81.6 | 7861 | 26.71 |
| d42-56 | 2 | 993 | 2666 | 2.69 | 75.6 | 6777 | 23.02 |
| d42-56 | 2 | 962 | 2789 | 2.90 | 77.0 | 7090 | 24.09 |
| d42-56 | 2 | 1057 | 3144 | 2.97 | 80.1 | 7981 | 27.74 |
| d42-56 | 3 | 1034 | 3212 | 3.11 | 79.5 | 8016 | 34.92 |
| d42-56 | 3 | 1037 | 2649 | 2.55 | 76.1 | 6610 | 28.79 |
| d42-56 | 3 | 923 | 2553 | 2.77 | 74.1 | 6372 | 27.75 |
| d42-56 | 3 | 964 | 3000 | 3.11 | 80.3 | 7488 | 32.62 |
| d42-56 | 4 | 1111 | 3167 | 2.85 | 85.8 | 8170 | 34.48 |
| d42-56 | 4 | 1037 | 2699 | 2.60 | 80.3 | 6963 | 29.39 |
| d42-56 | 4 | 822 | 2426 | 2.95 | 77.9 | 6258 | 26.41 |
| d42-56 | 4 | 1049 | 2939 | 2.80 | 84.1 | 7581 | 32.00 |
| d56-70 | 1 | 967 | 2964 | 3.07 | 92.5 | 7399 | 25.91 |
| d56-70 | 1 | 966 | 3402 | 3.52 | 95.0 | 8493 | 29.74 |
| d56-70 | 1 | 1003 | 3488 | 3.48 | 93.4 | 8707 | 30.49 |
| d56-70 | 1 | 856 | 2965 | 3.46 | 90.6 | 7400 | 25.91 |
| d56-70 | 2 | 887 | 3136 | 3.54 | 94.4 | 7973 | 27.09 |
| d56-70 | 2 | 864 | 2715 | 3.14 | 89.0 | 6901 | 23.45 |
| d56-70 | 2 | 909 | 2834 | 3.12 | 89.3 | 7205 | 24.48 |
| d56-70 | 2 | 994 | 3379 | 3.40 | 93.4 | 8591 | 29.19 |
| d56-70 | 3 | 1019 | 3298 | 3.24 | 92.8 | 8232 | 35.86 |
| d56-70 | 3 | 965 | 2892 | 3.00 | 90.6 | 7218 | 31.44 |
| d56-70 | 3 | 943 | 2776 | 2.94 | 86.4 | 6929 | 30.18 |
| d56-70 | 3 | 1046 | 3181 | 3.04 | 93.6 | 7937 | 34.57 |
| d56-70 | 4 | 970 | 3248 | 3.35 | 100.6 | 8379 | 35.36 |
| d56-70 | 4 | 867 | 2805 | 3.24 | 93.6 | 7236 | 30.54 |
| d56-70 | 4 | 959 | 3003 | 3.13 | 92.3 | 7747 | 32.70 |
| d56-70 | 4 | 1095 | 3374 | 3.08 | 99.1 | 8704 | 36.74 |
| d70-84 | 1 | 955 | 3066 | 3.21 | 105.5 | 7653 | 26.80 |
| d70-84 | 1 | 1091 | 3503 | 3.21 | 106.3 | 8745 | 30.62 |
| d70-84 | 1 | 964 | 3405 | 3.53 | 106.7 | 8500 | 29.76 |
| d70-84 | 1 | 1015 | 3192 | 3.14 | 102.0 | 7968 | 27.90 |
| d70-84 | 2 | 935 | 3409 | 3.65 | 107.0 | 8666 | 29.44 |
| d70-84 | 2 | 856 | 2310 | 2.70 | 101.3 | 5872 | 19.95 |
| d70-84 | 2 | 876 | 3111 | 3.55 | 102.2 | 7908 | 26.87 |
| d70-84 | 2 | 947 | 3408 | 3.60 | 106.2 | 8665 | 29.44 |
| d70-84 | 3 | 968 | 3460 | 3.58 | 105.6 | 8635 | 37.61 |
| d70-84 | 3 | 906 | 3290 | 3.63 | 103.2 | 8210 | 35.76 |
| d70-84 | 3 | 871 | 3036 | 3.48 | 98.0 | 7578 | 33.01 |
| d70-84 | 3 | 994 | 3678 | 3.70 | 108.5 | 9177 | 39.98 |
| d70-84 | 4 | 1060 | 3475 | 3.28 | 115.5 | 8963 | 37.83 |
| d70-84 | 4 | 829 | 2996 | 3.61 | 104.0 | 7729 | 32.62 |
| d70-84 | 4 | 823 | 3040 | 3.69 | 104.9 | 7842 | 33.10 |
| d70-84 | 4 | 766 | 3249 | 4.24 | 109.2 | 8382 | 35.38 |

^1^each observation is an average value of a pen (*n* = 8). In total, 196 observations were obtained.
